# Recent advances in electrochemical sensing and remediation technologies for ciprofloxacin

**DOI:** 10.1007/s11356-024-35852-9

**Published:** 2025-01-14

**Authors:** Vrinda Kini, Sreelakshmi C S, Debasmita Mondal, Nethaji Sundarabal, Pooja Nag, Kapil Sadani

**Affiliations:** 1 Department of Instrumentation and Control Engineering, Manipal Institute of Technology, Manipal Academy of Higher Education, Manipal, Karnataka India; 2 Department of Microbiology, Kasturba Medical College, Manipal Academy of Higher Education, Manipal, Karnataka India; 3 Department of Electrical and Instrumentation Engineering, Thapar Institute of Engineering and Technology, Patiala, Punjab India; 4 Department of Chemical Engineering, Manipal Institute of Technology, Manipal Academy of Higher Education, Manipal, Karnataka India; 5 Department of Mechatronics, Manipal Institute of Technology, Manipal Academy of Higher Education, Manipal, Karnataka India

**Keywords:** Electrochemical sensors, Substrates, Modifiers, Ciprofloxacin detection and remediation, Separation, Degradation

## Abstract

Ciprofloxacin (CIP) is an extensively used broad-spectrum, fluoroquinolone antibiotic used for treating diverse bacterial infections. Effluent treatment plants (ETPs) worldwide lack technologies to detect or remediate antibiotics. CIP reaches the aquatic phase primarily due to inappropriate disposal practices, lack of point-of-use sensing, and preloaded activated charcoal filter at ETPs. The co-existence of bacteria and CIP in such aqueous pools has promoted fluoroquinolone resistance in bacteria and should be minimized. The worldwide accepted standard detection methodologies for the detection of CIP are high-performance liquid chromatography and mass spectrometry, which are lab-based, require state-of-the-art equipment, and are expensive. Hence, it is difficult to integrate them for on-site monitoring. Further, the current remediation technologies like conventional sludge-treatment techniques fail to remove antibiotics such as CIP. Several point-of-use technologies for the detection of CIP are being investigated. These typically involve the development of electrochemical sensors where substrates, modifiers, biorecognition elements, and their chemistries are designed and optimized to enable robust, point-of-use detection of CIP. Similarly, remediation techniques like adsorption, membrane filtration, ion exchange, photocatalysis, ozonation, oxidation by Fenton’s reagent, and bioremediation are explored, but their onsite use is limited. The use of these sensing and remediation technologies in tandem is possibly the only way the issues related to antimicrobial resistance may be effectively tackled. This article provides a focused critical review on the recent advances in the development of such technologies, laying out the prospects and perspectives of their synergistic use to curb the menace of AMR and preserve antibiotics.

**Supplementary Information:**

The online version contains supplementary material available at 10.1007/s11356-024-35852-9.

## Introduction

Active pharmaceutical ingredients (APIs) are biologically active components used to treat diseases such as bacterial infections, hypertension, diabetes, inflammation, pain, cancer, and mental health disorders (Kumar et al. 2022b). Among APIs, the development, sensing, preservation, and remediation of antibiotics are becoming increasingly relevant in light of rampant antimicrobial resistance (AMR). Antibiotics are typically bactericidal or bacteriostatic. Fluoroquinolones are a class of bactericidal/bacteriostatic antibiotics which are commonly employed for their broad-spectrum activity. They reach the waterbodies through effluent treatment plants, which are not well equipped with detection and remediation technologies, persisting for a long period of time and causing conditions like antimicrobial resistance (AMR). A study conducted by Laxminarayan and Chaudhury (2016) found that *Escherichia coli* resistance to fluoroquinolones increased from 78 to 85% between 2008 and 2013, and *Salmonella typhi* isolates also showed an increase in fluoroquinolone resistance from 8% in 2008 to 28% in 2014 (Laxminarayan and Chaudhury 2016). Similarly, Dreyer et al. (2022) reported that 36% of multidrug-resistant *Mycobacterium tuberculosis* complex strains show fluoroquinolone resistance, contributing to high pre-extensive drug resistance in Mumbai, India (Dreyer et al. 2022).

Ciprofloxacin (CIP) is one of the most extensively used fluoroquinolone antibiotics employed for treating intra-abdominal infections caused by pathogens such as *Escherichia coli*, *Pseudomonas aeruginosa*, *Bacteroides fragilis*, *Proteus mirabilis*, and *Klebsiella pneumoniae* (Marchesini et al. 2007), skin infections, urinary tract infection (UTI), sexually transmitted diseases (STDs), and respiratory tract infections. However, CIP released into the environment through human excreta and inappropriate disposal of antibiotics is not completely metabolized, leading to the contamination of soil and water. Additionally, CIP has low biodegradability, high stability in water, and a hydrophilic nature, which makes it toxic to aquatic species (Kumar et al. 2022a). Food and Agriculture Organization of the United Nations has reported that it also inhibits the growth of freshwater producers like duckweed and cyanobacteria. Excessive exposure to CIP can cause damage to the central nervous system of animals and humans, among other major side effects (Gui et al. 2018). In Patancheru, an industrial area in Hyderabad, India, elevated levels of CIP were detected in wastewater treatment plant (WWTP) effluents up to 14 mg/L and from 2 to 6.5 mg/L in lakes (Rosas-Ramírez et al. 2022).

Following oral administration, 40 to 50% of the CIP is excreted in its original form through human excreta (Mahapatra et al. 2022), which reaches surface water through sewage or wastewater treatment plants. The co-existing antibiotics, their residues, and bacteria are causing an alarming amount of CIP resistance, making the drug less effective as a therapeutic. *B. anthracis*, *Pseudomonas aeruginosa*, *Neisseria* gonorrhoeae, *Enterococci*, *E*. *coli*, and *Klebsiella pneumoniae* are a few of the bacteria which are developing resistance to ciprofloxacin (Panhotra et al. 2004). Such conditions serve as a reservoir of ciprofloxacin with sub-lethal concentrations, thereby developing resistance towards the antibiotic. The proliferation and transfer of resistant genes to other bacteria (Patel et al. 2024) significantly increase the problems caused due to AMR, thereby reducing the effectiveness of standard therapies, increasing the healthcare cost, complicating the treatment for bacterial infections, and contributing to high mortality rates. Also, the diverse nature of APIs and their ability to resist the degradation process employed in an effluent treatment plant (ETP) pose challenges in complete removal. Additionally, the lack of point-of-use detection technologies in ETP has further affected the mitigation of APIs. It is thus important to limit the co-existence of ciprofloxacin with natural microbial fauna, necessitating the need to develop point-of-use technologies for the detection and remediation of CIP.

Worldwide accepted gold standard methods for CIP detection involve the tandem use of a modality of chromatography and mass spectroscopy such as high-performance liquid chromatography (HPLC) and high-resolution liquid chromatography mass spectrometry (HR-LCMS). Though highly accurate and sensitive, these require sophisticated analytical instrumentation facilities which are confined to state-of-the-art laboratories requiring operational expertise and are reagent extensive and expert comprehension with elaborate libraries. Biosensors are one of the technological solutions for the extensive need for antibiotic detection. To design a biosensor, a transduction platform must be used to detect the recognition event between the analyte of interest and the receptors present on the sensing substrate and transform it into a form that can be recorded. This transduction platform must be integrated with a biorecognition element that is specific to the target analyte. Antibodies (Pinacho et al. 2014), aptamers (Roushani et al. 2018), nucleic acids (Rowe et al. 2010), and enzymes (Wang et al. 2020) are the common biorecognition elements employed in antibiotic detection. Optical and electrochemical platforms are the most popular transduction platforms in the field of biosensing. Due to various advantages like low cost, less sample requirement, reduced analysis time, and simplicity due to ease of electronic configurations, electrochemical sensors are considered one of the best methods for detecting antibiotics in food and water (Khanmohammadi et al. 2020).

An electrochemical sensor is a powerful analytical tool which converts a chemical signal to an electrical signal through an electrochemical reaction. When designing an electrochemical sensor, screen-printed electrodes, glassy carbon electrodes, and microfabricated electrodes are the commonly used substrates. They are further altered with modifiers like graphene oxide (GO) (Pan et al. 2021), reduced graphene oxide (rGO) (Chauhan et al. 2020), metal sulfides (Santhiyagu Sahayaraj et al. 2023), metal oxides (Zhang et al. 2023), or metal nanoparticles (Amidi et al. 2017) to increase the electrocatalytic activity, facilitate simple electron transfer, and increase surface-to-volume ratio, thereby achieving higher sensitivity. The electrochemical oxidation of CIP is governed by both diffusion and kinetic processes (Shen et al. 2018). Additionally, at pH 7, ciprofloxacin exists in a zwitter ionic state and is found to be thermodynamically most reactive to electrochemical oxidation (Chaabani et al. 2022). These findings indicate the complex electrochemical behavior of ciprofloxacin and highlight its potential applications in electrochemical process-based applications. However, due to their high redox stability, detecting small molecules like ciprofloxacin requires the application of large overpotentials to initiate the redox reactions for sensing. Additionally, small molecule detection necessitates complex electrode modification to achieve selective and sensitive detection (Murugappan et al. 2022).

Remediation is a process of removing or catalyzing contaminants to non-hazardous products in the aqueous phase. These processes are tailored to mitigate antibiotics, like ciprofloxacin, present in the trace level in the environment. Different techniques such as adsorption (Mao et al. 2016), bioremediation (Girardi et al. 2011), and advanced oxidation processes (AOPs) (Lima et al. 2020) are employed in antibiotic removal. Adsorption employs materials such as activated carbon (Tran et al. 2022), multi-walled carbon nanotubes (Yu et al. 2016), clay minerals (Antonelli et al. 2020), and other materials to trap and remove the antibiotics. Whereas bioremediation utilizes microorganisms (Girardi et al. 2011) or plants (Sodhi et al. 2021) for effective removal of the antibiotics. Likewise, AOPs include techniques like photocatalysis (Yu et al. 2019) and ozonation (Sui et al. 2012), which employ reactive species in breaking down the antibiotic to its less harmful form. Effective remediation strategies can substantially help in the remediation of the antibiotics from the environment, thereby reducing the effect of antibiotics on human health and aquatic life. Hence, the widespread adoption of biosensors and remediation technologies holds a robust and ecologically balanced future. By prioritizing and practically applying these technologies, there can be a significant reduction of the contamination caused by ciprofloxacin, thus preserving CIP as an effective therapeutic and ensuring sustainable infectious disease management.

This review article presents a comprehensive study of the different types of electrochemical sensors and remediation strategies reported in the literature for the detection and remediation of ciprofloxacin. The article is organized as follows: the “Electrochemical sensor substrates” section discusses the various electrode materials implemented for robust detection of ciprofloxacin; the “Modifiers” and “Biorecognition elements” sections highlight the recent advances in various modifiers and receptors used for ciprofloxacin detection, and a detailed study on ciprofloxacin remediation is presented in the “Remediation techniques” section. Each section presents a critical literature review and lays out prospects and perspectives of the use of these technologies in tandem.

## Electrochemical sensor substrates

The development or use of an appropriate substrate is crucial in the development of an electrochemical sensor for ciprofloxacin detection. The choice of substrate influences the sensitivity, limit of detection (LoD), stability, reproducibility, and robustness of the sensor. Substrates typically constitute electrodes of gold, carbon, platinum, or other conductive material. The electrode serves as a platform for further modification with different modifiers and biorecognition elements specifically for interacting with the analyte. A critical insight into electrochemical substrates reported for ciprofloxacin detection is presented henceforth.

### Paper-based substrate

Paper is a flexible substrate developed from cellulose fibers, offering advantages such as flexibility, biodegradability, and microfluidic lateral flows, making them ideal eco-friendly disposable sensing substrates (Sadani et al. 2020). Cellulose-based substrates have inherent porosity and functional groups that facilitate modification with various modifiers such as carbon materials (de Souza et al. 2022), nano particles (Nilghaz and Lu 2019), and biorecognition elements (Chomthong et al. 2024) to enhance the sensitivity and selectivity of the sensor. Paper-based substrates consist of hydroxyl groups (-OH), which can be utilized to introduce carboxyl groups via oxidation, silane groups through silanization, amine groups through amination, and ester groups through the esterification process. In a study conducted by de Souza et al. (2022), a paper-based substrate was employed to detect ciprofloxacin using differential pulse voltammetry (DPV). The substrate was further modified using conductive ink, nail polish, and graphite powder. In this context, graphite may enhance the electrical conductivity of the substrate whereas nail polish can act as a binder. The authors achieved a detection limit of 4.96 µmol L^−1^. However, interference from other antibiotics present in the sample might affect the sensor output due to the lack of a biorecognition element. Also, the LoD does not meet the required maximum residue limits. Additionally, paper-based substrates pose challenges such as low electrical conductivity, variable surface property, mechanical fragility, and inconsistency in functionalization. Conductive materials like graphene, carbon nanotubes, or metal nanoparticles can be integrated with the substrate to resolve the issue of poor conductivity. Similarly, employing robust paper material in combination with polymer matrix can improve the durability of the substrate. To improve the consistency of functionalization, surface treatment methods such as chemical vapor deposition, UV treatment, electrochemical deposition, or silane coupling agents can be utilized. Additionally, precise control over the preparation and functionalization of the substrate is crucial to overcome the disadvantage of surface variability.

### Glassy carbon electrode (GCE)

**Fig. 1:**
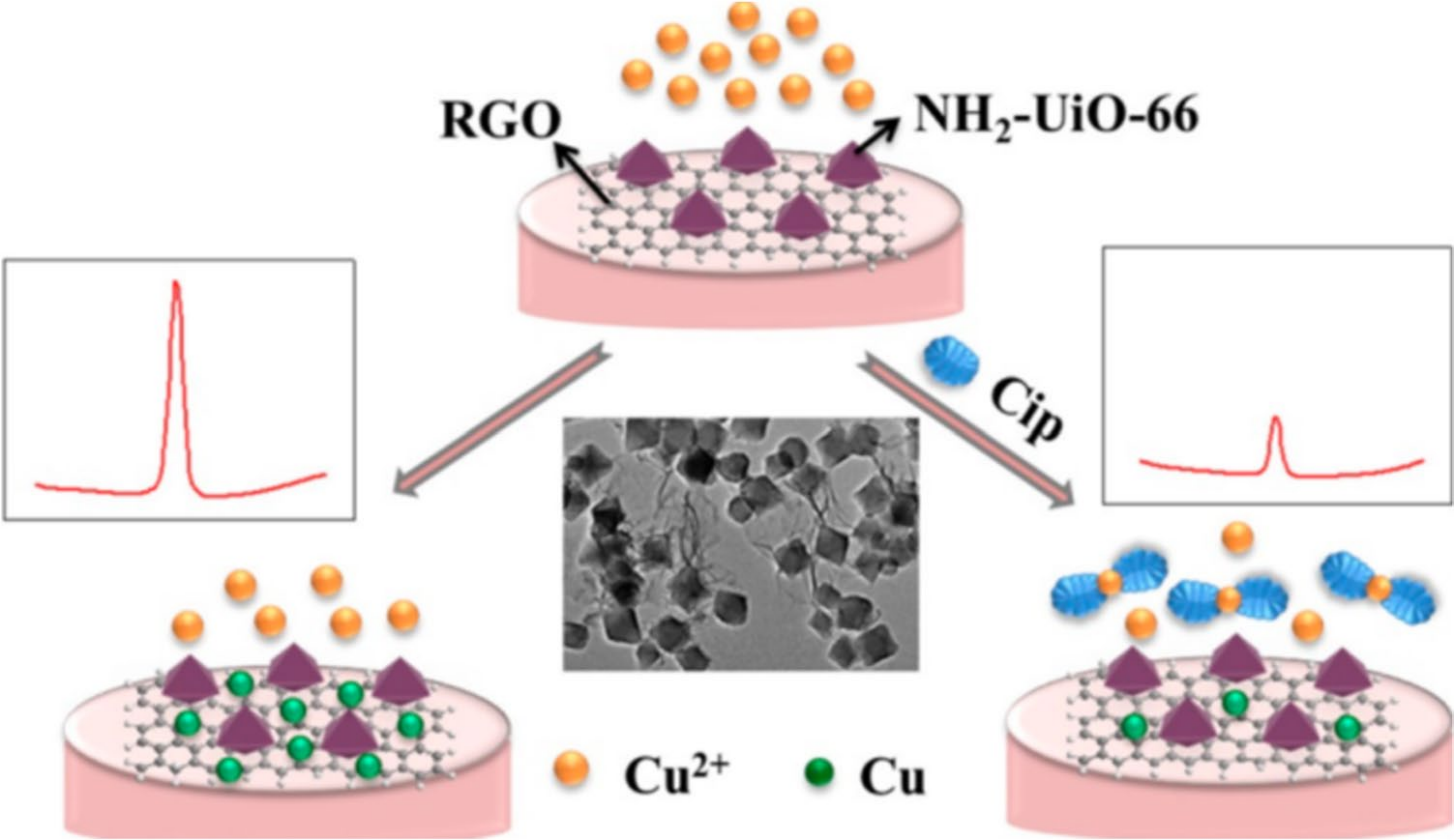
Schematics of ciprofloxacin detection using a glassy carbon electrode modified using rGO and NH_2_-UiO-66 (reprinted with permission from Fang et al. (2019), Copyright (2019), American Chemical Society)

Glassy carbon electrode (GCE) has been a popular choice of substrate in developing simple and rapid electrochemical ciprofloxacin sensors due to its electron transport characteristics, polarizable nature, stability, porosity, and large surface area (Wang et al. 2010b). The high pore volume of GCE facilitates easy modifications with catalytic nanomaterials and biorecognition elements. Fang et al. (2019) employed a GCE electrode modified with zirconium-based MOF to detect CIP in lake water (Fig. 1) using anodic stripping voltammetry. The authors demonstrated a detection limit of 6.67 nM. Though the sensor exhibited rapid and sensitive detection, a potential drawback might be its specificity when the concentration of the similar structured antibiotics exceeds that of the CIP concentration. Additionally, sensor fouling might need further research to increase the reliability and applicability of this technique in real-world scenarios. Similarly, in an interesting study conducted by Mariappan et al. (2023), ciprofloxacin was detected using cyclic voltammetry and differential pulse voltammetry in tap water and river water employing a GCE modified with a composite of ZnWO_4_ and carbon black, resulting in a LoD of 0.020 µM. However, when used for detection in a complex organic matrix containing a mixed antibiotic load, the fabricated sensor may not yield specific results for ciprofloxacin. Additionally, the technology needs to be investigated for matrix effects, and the packaging must be carefully revisited for point of use. GCE also has notable drawbacks such as surface fouling and very low catalytic activity. Surface fouling can be addressed by employing anti-fouling agents, whereas low catalytic activity can be resolved by modifying the electrode with nanomaterials like metal sulfides, metal oxide nanoparticles, graphene, or carbon nanotubes (CNTs). Additionally, proper handling and storage conditions including regular cleaning, maintaining dust-free environment, and ensuring the stability of surface modifications are necessary to address the fragility of the electrode.

Table 1 lists the details of some GCE-based electrochemical sensors reported in the literature for ciprofloxacin detection.

**Table 1:** Glassy carbon electrode-based sensors for electrochemical detection of ciprofloxacin

Sl. no	Analyte	Method	Sample	LOD	Remarks	Ref
1	Ciprofloxacin	Anodic stripping voltammetry using GCE modified with graphene and zirconium-based MOF Working electrode (WE): glassy carbon electrode The reference electrode (RE): Ag/AgCl saturated with KCl Counter electrode (CE): platinum wire	Tap water and lake water	6.67 nM in PBS^*#^	Due to the lack of a biorecognition element, there is a high chance that other antibiotics with similar structures will interfere with the sensor when it is exposed to a load of antibiotics	Fang et al. (2019)
2	Paracetamol and ciprofloxacin	Cyclic voltammetry using GCE modified with CMK-3, TiO_2_ sol, AuNPs, Nafion, and PB WE: GCE The reference electrode (RE): Ag/AgCl saturated with KCl Counter electrode (CE): platinum wire	River water and wastewater	Ciprofloxacin, 1.08 × 10^−1^ µM; paracetamol, 0.21 µM in environmental water^*^	Simultaneous detection of two small molecules on the same electrode might lead to fouling of the electrode, hence affecting its performance	Pollap et al. (2020)
3	Ciprofloxacin	Differential pulse voltammetry using GCE modified with activated carbon. AuNPs and supramolecular solvent WE: GCE CE: platinum electrode RE: Ag/AgCl electrode	Milk and tablets	0.20 nM in PBS^*^	The sensor can be expected to have better selectivity towards the analyte if a biorecognition element is employed	Gissawong et al. (2021)
4	Ciprofloxacin	Differential pulse voltammetry using GCE modified with functionalized multi-walled carbon nanotubes (MWCNTs) and polydopamine WE: GCE CE: platinum electrode RE: Ag/AgCl electrode	Plasma and urine	4.00 × 10^−2^ µM in phosphate buffer^*^	Poor LoD and lack of cross-sensitivity studies with similarly structured antibiotics restrict the use of the sensor for biological samples only	Sabeti et al. (2021)
5	Ciprofloxacin	Cyclic voltammetry and differential pulse voltammetry using GCE modified using the composite of ZnWO_4_ and carbon black material. The reference electrode (RE): Ag/Agcl saturated with KCl Counter electrode (CE): platinum wire	Tap water and river water	2.00 µ × 10^−2^ M in PBS^*^	Interference from other antibiotics present in the sample might affect the sensor output due to the lack of a biorecognition element Also, the LoD does not meet the required maximum residue limits	Mariappan et al. (2023)
6	Ciprofloxacin	Voltametric detection using GCE modified with Cu-MOF doped with ruthenium WE: GCE CE: platinum electrode RE: Ag/AgCl electrode	Tap water and seawater	3.29 nM in phosphate buffer^*^	Interference issue due to the absence of specific bio-receptors	Varsha and Nageswaran (2023)
7	Ciprofloxacin	Electrochemical detection using glassy carbon electrode modified with poly 2‑(hydroxymethyl)thiophene WE: GCE CE: platinum electrode RE: Ag/AgCl electrode	Human urine	0.10–2.00 × 10^2^ µM in citrate buffer^*^	Poor LoD and lack of cross-sensitivity studies with similarly structured antibiotics restrict the use of the sensor for biological samples only	Burç et al. (2023)
8	Ciprofloxacin	Electrochemical detection using glassy carbon electrode modified with gold nanoparticles, S-CoFe-MOFs, and molecularly imprinted polymers (MIP) WE: GCE CE: platinum electrode RE: Ag/AgCl electrode	Milk	3.30 × 10^−6^ µg/mL^*^	The cross-sensitivity of this system needs to be better established for point-of-use applications	Xiong et al. (2023)
9	Ciprofloxacin	Electrochemical detection using glassy carbon electrode (GCE) modified with MnO_2_/ZnO WE: GCE CE: platinum electrode RE: Calomel electrode	Honey	0.21 µM in PBS^*^	Due to poor LoD and a lack of a biorecognition element, the sensor performance may be hampered when exposed to an antibiotic load	Zhang et al. (2023)

In the table, the LoDs have been determined using the following notations: ^*#^signal to noise ratio = 3, ^*^3 *σ*/*m*, where *σ* is the standard deviation and *m* is the slope

*S/N* signal to noise ratio, *σ* standard deviation, *m* slope

### Screen-printed electrode (SPE)

**Fig. 2:**
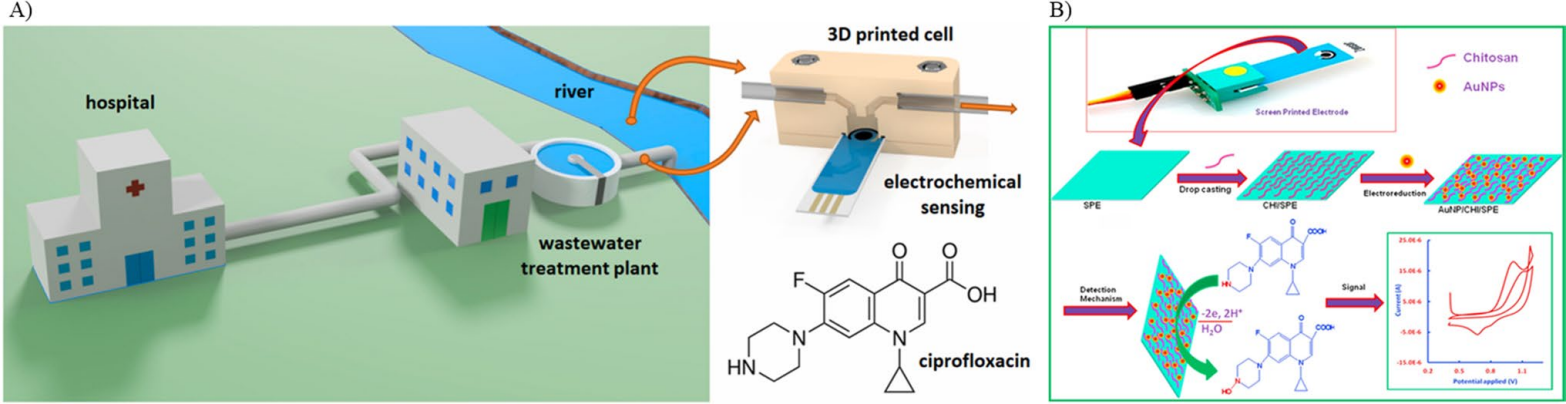
**A** Schematics of ciprofloxacin detection using a boron-doped diamond screen-printed electrode modified (reprinted with permission from Dakošová et al. (2023), Copyright (2023), Elsevier). **B** Schematics of ciprofloxacin detection using a disposable screen-printed electrode modified with composite film of gold nanoparticle and chitosan (reprinted with permission from Reddy et al. (2018), Copyright (2018), Elsevier)

Screen-printed electrodes (SPE) offer great potential for point-of-care applications and hence have garnered popularity over the years. It offers various advantages, including low cost, high reproducibility, and ease of use. The working electrode undergoes modification with different nanomaterials and biorecognition elements to enhance the sensitivity and selectivity, respectively, of the sensing process. In a study by Dakosova et al*.* in 2022, CIP was detected in a wastewater treatment plant using an electrochemical flow-through. Boron-doped diamond SPE was used as the sensor substrate, and analysis of CIP was done through cyclic voltammetry (CV) and square wave voltammetry (SWV) techniques (Dakošová et al. 2023). Figure 2A illustrates the sensing scheme employed in this research. While detection of CIP using the sensor presents a promising possibility of matrix effects and cross-sensitivity with compounds having similar redox peaks, it may present potential limitations while monitoring the antibiotic residues in sewage systems. Additionally, sewage water may contain harmful chemicals that can lead to the biofouling of electrodes. This biofouling causes the electrode surface to become passivated by fouling agents, forming impermeable layers. These layers limit the interaction between the analyte and the electrode surface, hindering the electron transfer (Cinti and Arduini 2017). Wong et al*.* (2023) detected CIP in river water and simulated urine samples. SPE was used as the sensor substrate, which was modified with carbon black and Fe_3_O_4_ nanoparticles. Further, a molecular imprinted polymer (MIP) specific to the target analyte was employed as a recognition element. Electrochemical analysis of ciprofloxacin was carried out using CV and differential pulse voltammetry (DPV) which produced a linear response in the range of 0.50–7.00 µmol/L (Wong et al. 2023). Although the sensor achieved a lower limit of detection of 8.40 × 10^−3^ µmol L^−1^, the stability of the electrode over time and across different real samples might need further investigation. Although SPEs are commonly used in electrochemical sensing, they face several challenges. One of the disadvantages of SPE is reproducibility, where the printing process can create inconsistencies in the morphology, uniformity, and thickness, thereby effecting the sensor performance. To address this issue, precise control over the printing technology is necessary. Additionally, SPEs are easily prone to fouling by the sample; hence, they can be protected by using anti-fouling agents. The inherent low conducting property of the carbon used in the printing process can also impact the sensing process. This can be resolved by integrating the SPE with graphene, metal nanoparticles, or carbon nanotubes. Similarly, chemical stability of the ink may degrade with time in various environments. To overcome this issue, chemically inert inks can be utilized. Similarly, Reddy et al. (2018) employed a composite film of gold nanoparticles and chitosan to modify the disposable screen-printed electrode for CIP detection (Fig. 2B) Square wave voltammetry technique was used for the detection. The authors achieved a detection limit of 1.00 × 10^−3^ µM. The use of biorecognition elements may provide higher specificity to target antibiotics.

Table 2 presents a list of various screen-printed electrode-based biosensors employed for ciprofloxacin detection.

**Table 2:** Recent advances in screen-printed electrodes in electrochemical detection of ciprofloxacin

Sl. no	Analyte	Method	Sample	LOD	Remarks	Ref
1	Ciprofloxacin	Square wave voltammetry using a screen-printed electrode modified with chitosan-gold nanoparticle (AuNp/CHI) composite film	Serum, plasma, and urine samples	1.00 × 10^−3^ µM in phosphate buffer^*^	The use of biorecognition elements may provide higher specificity to target antibiotics	Reddy et al. (2018)
2	Ciprofloxacin	Linear sweep voltammetry using oxygen-terminated and hydrogen-terminated boron-doped diamond powder (BDDP) printed electrodes	Diluted artificial urine	8.98 × 10^−1^ µM in PBS^*^	Diluting the urine sample a hundred times may affect the sensitivity of the sensor	Matsunaga et al. (2020)
3	Ciprofloxacin	Cyclic voltammetry, electrochemical impedance spectroscopy, and square wave voltammetry using screen-printed carbon electrodes modified with carboxylated carbon nanotubes	Spiked water samples	1.52 nM in PBS^#^	Maintaining the stability and functionality of DNA Gyrase enzyme during detection may be challenging	Cardoso et al. (2021)
4	Ciprofloxacin	Differential pulse anodic stripping voltammetry (DPSV) using screen-printed carbon electrode (SPCE) modified with graphene	Milk sample	0.30 µM in B-R buffer^*#^	The selection of appropriate biorecognition elements can mitigate the effect of other interferences in milk samples	Pan et al. (2021)
5	Ciprofloxacin	Differential pulse voltammetry using screen-printed electrode modified with vanadium pentoxide (V_2_O_5_) nanoparticles	CIP tablet and urine samples	1.00 × 10^−2^ µM in PBS	A more sophisticated deposition technique may be used to deposit nanoparticles to avoid poor adhesion	Tajik et al. (2021)
6	Ciprofloxacin	Square wave voltammetry using choline chloride-modified carbon paste electrode (ChCl/CPE)	Egg samples, CPRO eye drops, and river water samples	0.36 nM in citrate buffer^*^	The sensor is not portable and not capable of on-site analysis	Adane et al. (2023)
7	Ciprofloxacin	Differential pulse voltammetry using screen-printed electrode modified with Fe_3_O_4_ magnetic nanoparticles coated with MIP and carbon black	Synthetic urine and river water samples	8.40 × 10^−3^ µM in Britton-Robinson buffer^*^	MIPs designed for selectivity may show cross-reactivity with similar molecules	Wong et al. (2023)
8	Ciprofloxacin and methotrexate (MTX)	Square wave voltammetry using a screen-printed electrode modified with nanocellulose, 3-dimensional polypyrrole (NC-3DPPY), and silver-gold (Ag-Au) bimetallic alloy nanocomposite	Tablets were dissolved in PBS, and blood serum samples and water samples were simulated	6.20 × 10^−2^ µM (MTX) 0.17 × 10^−1^ µM (CIP) in PBS^*^	Simultaneous detection of two antibiotics may lead to electrode fouling, further reducing the sensitivity of the sensor	Akhter et al. (2022)

In the table, the LoDs have been determined using the following notations: ^*^3 *σ*/*m*, ^#^*X* + 3*σ*, where *σ* is the standard deviation, *m* is the slope, and *X* is the average of blank signals

### Microfabricated substrates

Microfabricated electrochemical substrates refer to the miniature structures manufactured through the microfabrication process. It involves clean room procedures like cleaning, oxidation, lithography, etching, and other steps. Microfabrication enables the development of electrochemical substrates of dimensions ranging from a few micrometers to nanometers, thus increasing the surface area and sensitivity. It further decreases the sample volume. Kim et al. (2009) detected oxytetracycline using gold interdigitated array (IDA) modified with ssDNA. Similarly, Xu et al. (2016), employed interdigitated array microelectrodes (IDAMs) modified with antimony tin oxide nanoparticles (nano ATO) and chitosan. Aptamer was immobilized on the modified IDAM. In this study, chitosan was employed to disperse the nano ATOs uniformly and to fix them firmly on the IDAM. Whereas the nano ATOs were employed to promote the electron transfer and to enhance the electrochemical signal. The authors achieved a detection limit of 3.00 × 10^−9^ g/mL. Despite the advancements in antibiotic detection in these substrates over the past two decades (Hong et al. 2011), the field remains unexplored in ciprofloxacin detection. The use of microfabricated sensing systems provides easy scalability limiting the need for frequent baselining and sensitivity drifts. However, they face challenges such as complex fabrication process and stability issue. The cost and the complexity of fabrication can be mitigated by employing simple, cost-effective techniques such as inkjet and screen-printing technologies. Stability problems can be addressed by introducing protective layers like polymeric coating, silica-based coating, or others.

## Modifiers

**Fig. 3:**
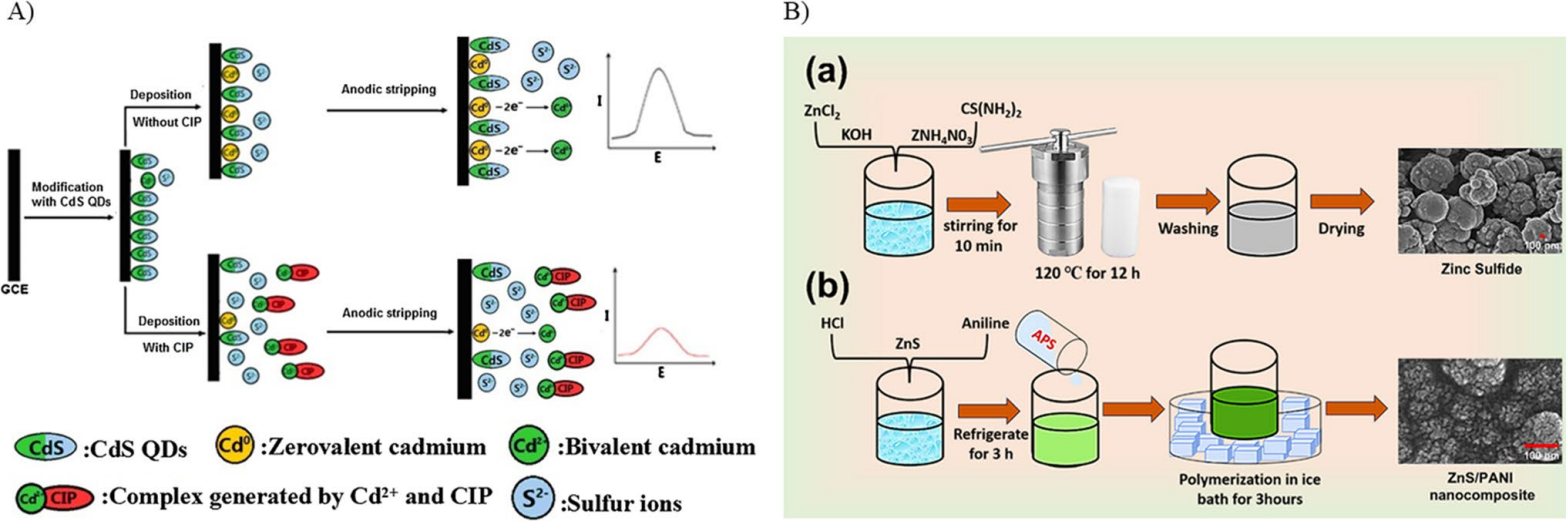
**A** Schematics of ciprofloxacin detection using quantum dots as a modifier (reprinted with permission from Shan et al. (2016), Copyright (2016), Elsevier). **B** Schematics of electrochemical detection of ciprofloxacin using metal sulfide as a modifier (reprinted with permission from Ali et al. (2023), Copyright (2023), Springer Nature)

Electrochemical substrates are further modified using suitable nanostructured modifiers such as graphene, metal sulfides, metal oxides, carbon nanotubes, metal–organic frameworks, and covalent organic frameworks to enhance the charge transfer characteristics or render a definitive catalytic activity useful for the detection of ciprofloxacin (Tables 3, 4, and 5). Nanostructured modifiers with good conductivity and thermal and chemical stability are preferred as suitable modifiers in electrochemical sensing. Initially, CNTs (Ensafi et al. 2010) were one of the most explored materials, followed by metal oxide nanoparticles (Ensafi et al. 2012), graphene (Zhang et al. 2014), quantum dots (Shan et al. 2016), MOFs (Fang et al. 2019), and COFs (Zhu et al. 2020). Also, metal sulfides (Ali et al. 2023) have been introduced as a modifier for CIP detection (Fig. 3B).

**Table 3:** Graphene

Sl. no	Analyte	Method	Sample	LoD	Remarks	Ref
1	Ciprofloxacin	Cyclic voltammetry and differential pulse voltammetry using alizarin red and graphene-modified glassy carbon electrode	Human serum and tablet samples	1.00 × 10^−2^ µM in PBS^*#^	The lack of a biorecognition element can lead to interference from other antibiotics in the sample	Zhang et al. (2014)
2	Ciprofloxacin	Cyclic voltammetry using graphene-modified glassy carbon electrode	Ciprofloxacin is ciprofloxacin hydrochloric drugs	2.00 × 10^−2^ µmol/L.^*#^	The lack of a biorecognition element can lead to interference from other antibiotics in the sample	Xie et al. (2015)
3	Ciprofloxacin	Square wave voltametric detection using graphene-modified glassy carbon electrode coated with salmon sperm dsDNA as bioreceptor	Diluted human serum and urine	0.10 µM in acetate buffer^**^	The presence of any electrical screening substance in the sample can lead to interference in the electrostatic interaction between the DNA and CIP	Lim and Ahmed (2016)
4	Paracetamol and ciprofloxacin	Differential pulse voltammetry using glassy carbon electrode modified with GO and nickel nanoparticles	Urine and serum samples	Paracetamol, 6.70 nmol/L; ciprofloxacin, 6.00 nmol/L in phosphate buffer^*^	Since no biorecognition element was used, the sensor performance might be affected in the presence of interferences	Martin Santos et al. (2017)
5	Ciprofloxacin	Differential pulse voltammetry using GCE modified with rGO functionalized with gold nanoparticle-coated beta-cyclodextrin	Tap water	2.70 nM in PBS	Cross-sensitivity studies with similarly structured antibiotics should be conducted	Pham et al. (2018)
6	Chloramphenicol and ciprofloxacin	Cyclic voltammetry using glassy carbon electrode modified with graphene, carbon nitride, and gold nanoparticles	Milk samples	Chloramphenicol, 2.70 × 10^−8^ M; ciprofloxacin, 4.20 × 10^−7^ M in phosphate buffer^*#^	Simultaneous detection of antibiotics might hinder the sensor performance due to fouling	Yuan et al. (2018)

In the table, the LoDs have been determined using the following notations: ^*#^signal to noise ratio = 3, ^*^3 *σ*/*m*, and ^**^experimental LoD, where *σ* is the standard deviation and *m* is the slope

**Table 4:** Metal oxide nanoparticles

Sl. no	Analyte	Method	Sample	LOD	Remarks	Ref
1	Ciprofloxacin	Cyclic voltammetry using GCE modified with Ba_0.5_Co_0.5_Fe_2_O_4_ nanoparticles	Tablet	5.80 × 10^−9^ nM in PBS	When exposed to an antibiotic load, the sensor might cross-react with similarly structured antibiotics	Osman et al. (2015)
2	Ciprofloxacin	Differential pulse voltammetry using GCE modified with CoFe_2_O_4_-MWCNT	NA	3.60 × 10^−2^ µM in Britton-Robinson buffer^*^	Real sample analysis should have been conducted	Hosseini et al. (2019)
3	Ciprofloxacin	Differential pulse voltammetry using glassy carbon electrode modified with polyethyleneimine/Fe_3_O_4_ nanoparticles/carbon nanotubes	Pharmaceutical samples, urine, and serum	3.00 × 10^−3^ µmol L^−1^ in Britton-Robinson buffer^*^	Cross-sensitivity studies with similarly structured antibiotics were not conducted	Jalal et al. (2019)
4	Ciprofloxacin	Differential pulse voltammetry using screen-printed electrode modified with V_2_O_5_ nanoparticles	Urine and tablet	1.00 × 10^−2^ µM in PBS	Cross-sensitivity studies were not conducted	Tajik et al. (2021)
5	Ciprofloxacin	Differential pulse voltammetry using ITO-coated glass modified with lanthanum oxide nanoparticles	Milk	1.00 × 10^−3^ ng mL^−1^ in PBS^*^	Further sensitivity improvement can be achieved using other nanomaterials like graphene and nanotubes, combined with the present material	Chaudhary et al. (2021)
6	Ciprofloxacin	Cyclic voltammetry using GCE modified with MgFe_2_O_4_-MWCNTs	Urine, plasma, and tablet	1.00 × 10^−2^ µmol L^−1^ in PBS^#^	Interference studies of the sensor performance should have been conducted with similarly structured antibiotics	Ensafi et al. (2012)

In the table, the LoDs have been determined using the following notations: ^*^3 *σ*/*m*, ^#^*X* + 3*σ*, where *σ* is the standard deviation, *m* is the slope, and *X* is the average of blank signals

**Table 5:** carbon nanotubes

Sl. no	Analyte	Method	Sample	LoD	Remarks	Ref
1	Ciprofloxacin	DPV Boron-doped diamond electrode modified with porous Nafion film and multi-walled carbon nanotubes	Water and wastewater samples	5.00 × 10^−2^ µM in KH_2_PO_4_^##^	The use of biorecognition elements may enhance the specificity of ciprofloxacin detection	Gayen and Chaplin (2016)
2	Ciprofloxacin	CV Glassy carbon electrode modified with polyaniline film and multi-walled carbon nanotubes-beta cyclodextrin composite	Wastewater treatment plant effluent	5.00 × 10^1^ nM in PBS^*^	Glassy carbon electrodes are relatively expensive	Garrido et al. (2017)
3	Ciprofloxacin	DPV Glassy carbon electrode (GCE) modified with nanocomposite comprising of multi-walled carbon nanotubes, magnetite nanoparticles, and polyethyleneimine	Urine, commercial tablet and serum samples	3.00 × 10^−3^ micro mol/L in Britton-Robinson buffer^*^	Although GCE is a widely used electrode, it is impractical in portable setups and unsuitable as a disposable or semi-disposable sensor substrate in point-of-use or point-of-care applications	Jalal et al. (2019)
4	Ciprofloxacin	EIS and CV The screen-printed carbon electrode was modified with a nanocomposite comprising multi-walled carbon nanotubes, vanadium oxide, and chitosan Electrodes were functionalized with CPX ssDNA aptamer using EDC-NHS linker chemistry	Spiked milk samples	0.50 ng/mL in PBS^*/^	Degradation of aptamers by various enzymes present within samples, especially biological samples, limits their practical application with real samples	Hu et al. (2018)
5	Ciprofloxacin and paracetamol	EIS, CV, SWV Screen printed electrode modified with nanocellulose-polypyrrole matrix and single-walled carbon nanotubes	Water, biological fluids, pharmaceutical samples	Paracetamol, 7.20 × 10^−2^ nM; ciprofloxacin, 0.196 nM in PBS^*#^	Simultaneous detection of multiple analytes present in biological fluids can result in biofouling of electrodes	Shalauddin et al. (2022)

In the table, the LoDs have been determined using the following notations: ^*#^signal to noise ratio = 3, ^*^3 *σ*/*m*, ^##^signal to noise ratio is ≥ 5, ^*/^3*σ*, where *σ* is the standard deviation and *m* is the slope

This section and SI-2 discuss the various modifiers reported in the literature for the sensitive detection of ciprofloxacin.

### Carbon nanotubes

**Fig. 4:**
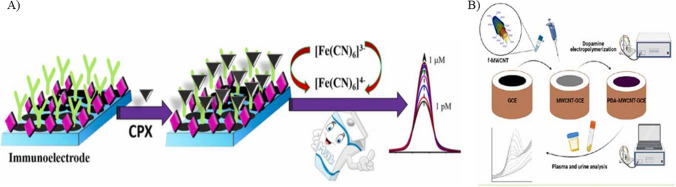
**A** Schematics of electrochemical detection of ciprofloxacin using metal oxide as a modifier (reprinted with permission from Chaudhary et al. (2024), Copyright (2024), Elsevier). **B** Schematics of electrochemical detection of ciprofloxacin using carbon nanotube as a modifier (reprinted with permission from Sabeti et al. (2021), Copyright (2021), IEEE Sensors Journal)

Carbon nanotube is one of the most used materials owing to its novel mechanical, electrical, and thermal properties (Khoshsafar et al. 2016). A study by Garrido et al. (2017) detected CIP in wastewater effluents using GCE as the sensor substrate, which was further modified to a composite consisting of polyaniline, β-cyclodextrin, and functionalized MWCNTs. It was observed that ciprofloxacin in acidic pH exists as a zwitter ion and shows a tendency to bind to β-cyclodextrin rather than to the edges of MWCNTs. Functionalized MWCNTs repelled the anionic group on ciprofloxacin, which enhanced the overall sensitivity of ciprofloxacin detection. The sensor detected ciprofloxacin to a detection limit of 5.00 × 10^1^ nM. In another study, Sabeti et al. (2021) detected ciprofloxacin in plasma and urine samples using modified GCE for therapeutic drug monitoring. The GCE was modified with functionalized MWCNTs and polydopamine, as shown in Fig. 4B. Polydopamine was employed to increase the electrode’s active surface area and also to facilitate the rate of electron transfer. The detection of ciprofloxacin was achieved using CV and DPV, and the sensor showed a LoD of 4.00 × 10^−2^ µM. However, the complex process involved in synthesizing and modifying using MWCNTs may be limited by automation capabilities for mass fabrication. This limitation can be overcome through process optimization and standardization. Table 5 presents recent studies on biosensors that employ carbon nanotubes as electrode modifiers for detecting ciprofloxacin.

### Metal oxide nanoparticles

Metal oxide nanoparticles consist of distinctive photochemical and electronic properties (Katz et al. 2004). Metal oxide nanoparticles are employed to modify the working electrode (Bagheri et al. 2016) utilizing a variety of methods, including electro-polymerization, electrodeposition, physical adsorption, and covalent chemical bonding (Nag et al. 2024b; Wang and Hu 2009). The versatility of metal oxide nanoparticles in modifying the working electrode contributes to advancements in sensor design technologies (Sadani et al. 2017). Ensafi et al. (2012) detected a ciprofloxacin sensor using GCE modified with MgFe_2_O_4_ and multi-walled carbon nanotubes. A precursor for MgFe_2_O_4_-MWCNTs was synthesized using the sol–gel method. Both Mg and Fe present in the composite are electrocatalytic in nature due to their electronic arrangement, facilitating increased electron transfer, thereby resulting in a current response which is proportion to the concentration of ciprofloxacin. The authors achieved a LoD of 1.00 × 10^−2^ µmol L^−1^, but the sensor performance in the presence of similar structured interfering antibiotics was not studied, and hence, its end use in real-world conditions cannot be ascertained. Another study conducted by Chaudhary et al. (2021) detected ciprofloxacin in milk using indium tin oxide (ITO) electrodes, modified with lanthanum oxide nanoparticles and antibody as depicted in Fig. 4A. Wet-chemical co-precipitation method was employed to synthesize the lanthanum oxide nanoparticles. Lanthanum oxide nanoparticles were employed because of their unique electrochemical property, where they transfer electron within a 4f shell, which helps in enhancing the conductivity. It also consists of numerous free active sites for binding, which enables increased immobilization of the antibody, by functionalizing it with APTES and using EDC-NHS linker. The detection limit of the sensor was 1.00 × 10^−3^ ng/mL. The employed nanomaterials and the antibody might degrade with time; hence, ensuring the long-term stability of the sensor is crucial for maintaining its performance over an extended period. Table 4 presents a list of sensors using various metal oxide nanoparticles as modifiers.

### Graphene

Graphene is made up of a monolayer of sp^2^ hybridized carbon atoms arranged in a hexagonal lattice. It facilitates the immobilization of the sensing materials, improving the sensitivity of the sensor. Furthermore, the electronic mobility of graphene increases charge transfer, enhancing the sensor’s overall performance. In a study conducted by Zhang et al. (2014), a GCE was modified with graphene and poly(alizarin red). Graphene enhances conductivity and also increases the surface-to-volume ratio, whereas poly(alizarin red) serves as a proton receptor facilitating a balanced reaction and promoting the transfer of electrons in between the electrode and the analyte. The lack of a biorecognition element can lead to interference from other antibiotics when employed in complex matrices. Hence, further analysis of matrix effects and sensor packaging may render this technology deployable for real-time monitoring. Similarly, Sawkar et al. (2022) detected ciprofloxacin by modifying a carbon paste electrode using graphene and sodium dodecyl sulfate. Sodium dodecyl sulfate being an anionic surfactant promotes electron transfer and increases the reaction rate between the electrode and the analyte. A LoD of 2.90 × 10^1^ µM was demonstrated. However, due to the absence of a biorecognition element, the complexity of the matrix in the real-world scenario may affect the specificity of the sensor. Table 3 lists the details of some graphene-based electrochemical sensors reported in the literature for ciprofloxacin detection.

### Quantum dots

Quantum dots (QDs) are nanomaterials with great potential in electrochemical sensing due to their electronic properties such as quantum confinement and tunable band gaps. They also exhibit unique optical properties like high quantum yield and photoluminescence. The effect of quantum confinement in quantum dots is crucial, where the energy levels are quantized, leading to tunable bandgaps which can be controlled by tuning the size of the quantum dots. The electron density is increased at the surface due to the high surface-to-volume ratio, offering multiple active sites for the transfer of electrons and increasing the total conductivity and sensitivity of the material. The excellent tunable optical properties of quantum dots have paved the way for the development of various optical sensors (Chullasat et al. 2018; Cotta 2020). QDs-based sensors are highly precise and selective, making them suitable for detecting different analytes (Karadurmus et al. 2021). In a study conducted by Shan et al. (2016), a GCE was further modified with cadmium sulfide QDs (CdS QDs) for the detection of CIP. The modified electrode displayed an anodic stripping signal due to the generation of Cd (II) ions from the QDs (Fig. 3A), achieving a detection limit of 2.20 × 10^−8^ mol L^−1^. Cd (II) ions are released from the QDs when in contact with ciprofloxacin due to an affinity-driven reaction. This interaction is crucial in anodic stripping voltammetry since the ions are stripped from the electrode, in turn enabling sensitive detection. Other heavy metals may interact distinctively with the surface of the electrode in electrochemical sensing by forming complexes or by undergoing redox reaction, when specific modifications and conditions are provided. However, interference caused by the other organic fluorescent components present in the complex samples may hinder the sensor performance by influencing the accuracy and selectivity of the sensor. Moreover, quantum dots are susceptible to degradation and photo-oxidation, which may decline the sensor performance over time. The complexity of the electrode modification is crucial in terms of cost-effectiveness and scalability. Considering the hazardous environmental effects of cadmium is also crucial. Similarly, in another study, a GCE modified with carbon QDs, ZnO nanoflowers, and poly(cetyltrimethylammonium bromide) achieved a detection limit of 1.97 nM (Hatamluyi et al. 2020). Though QDs as electrochemical sensing materials for CIP detection are promising, addressing challenges related to the interferences due to biomolecules and redox actives species in biological samples, long-term stability, and scalability is challenging for successfully translating this method into practical applications.

### Metal–organic frameworks (MOFs)

Metal–organic frameworks (MOFs) contain a charged metal ion enclosed by organic molecules, developing a highly regular, porous structure with a high surface area resembling a cage-like network. MOFs such as ZIF-8 (Hu et al. 2021), UIO-66 (Weng et al. 2022), HKUST-1 (Liu et al. 2024), Ni-MOF (Lv et al. 2022), Co-MOF (Shi et al. 2023), and Fe-MOF (Saeb and Asadpour-Zeynali 2022) are the commonly used MOFs in antibiotic detection. Organic molecules such as the N-donor group, phosphonates, and carboxylates are employed. Their flexibility in structure, porosity, and tailoring ability with respect to different functional groups have made the material successful for various applications (Giménez-Marqués et al. 2016; Wang 2017). Composite structures of MOFs have been produced to overcome the problem of the fragility of the functional groups present in the MOFs (Zhu and Xu 2014; Kempahanumakkagari et al. 2018). This results in good mechanical stability, better catalytic performance, and better conductivity. In the study conducted by Fang et al. (2019), a zirconium (Zr)-based MOF-modified electrode was used with rGO in detecting ciprofloxacin in water samples. Coordination chemistry of zirconium helps in the formation and functionality of the MOF. Zirconium ions behave like nodes which connects with 2-aminoterephthalic forming Zr-O bonds, developing a porous structure. Additionally, due to its large surface area, it provides multiple active sites for analyte detection. Further deposition of Cu^2+^ on the modified electrode leads to the complexation reaction forming Cu^2+^-CIP complex, thereby decreasing the oxidation current of Cu^2+^. The sensor detected ciprofloxacin up to 6.67 nM. In another study, Varsha and Nageswaran (2023) synthesized copper-based MOF as a modifier, which was further doped with ruthenium metal ion to increase the number of electrocatalytic active sites that can interact with CIP. The authors detected ciprofloxacin up to 3.29 nM. Though the sensor demonstrated an acceptable LoD, the synthesis of MOF needs precise control over the experimental condition, further affecting the scalability and reproducibility of the sensor for large-scale applications. Additionally, MOF might be unstable in harsh conditions, such as extreme pH levels and high humidity, which can influence the sensor performance. However, field use of MOFs in sensing and remediation technologies remains limited.

### Covalent organic frameworks

Covalent organic frameworks (COFs) are crystalline polymeric materials, synthesized via solvothermal and ionothermal methods, defined by excellent stability and long-range order. They are formed through the direct linkage of organic units via covalent bonding by eliminating the need for a metallic ion, thereby improving the stability, reducing the toxicity, and enhancing the biocompatibility of the material. COFs also display tunable pore size, good loading capacity, high thermal stability, and lower density. In a study conducted by Zhu et al. (2020), an electrochemical aptasensor with Au-electrode as a substrate for selective detection of CIP has been reported. The electrode was further modified with a COF synthesized using 1,3,5-tris(4-aminophenyl)benzene and 2,5-dimethoxyterephaldehyde confined with the gold nanoparticles (Au@COF). Further, the aptamer was immobilized on the modified electrode. Au@COFs provide good surface area, porosity, and stability and also provide numerous π-functional sites for immobilization of the aptamer. The authors achieved a detection limit of 2.34 fg/mL using the electrochemical impedance spectroscopy technique. However, the presence of nucleases in the complex environmental samples may degrade the aptamer over time, which might, in turn, affect the reliability and stability of the sensor.

### Metal sulfides

Metal sulfides are semiconducting electrode materials composed of sulfur anions and metal cations. They possess several novel properties such as conductivity, redox-reversibility, capacitance (Kulkarni et al. 2017), catalytic and photocatalytic activity, and optical characteristics (Jamal et al. 2023). Additionally, sulfur can stabilize multiple oxidation states of the metal to form a stable metal-sulfur bond. Metal sulfides are generally categorized as semiconductors, and a few of them exhibit conducting behavior, such as NbS_2_ (Chhowalla et al. 2013), while others show insulating behavior, like HFS_2_. Metal sulfides such as ZnS and CuS act as semiconducting materials, with band gap energies of 3.7 eV and 1.2 eV, respectively. Further, doping approaches can help improve conductivity, which increases with the concentration of the dopant. This increase in conductivity is due to the presence of excess free carriers (Lai et al. 2012). Thus, metal sulfides are widely employed in electrochemical biosensors as they can be easily synthesized in situ using physical and chemical methods and can be directly grown on substrates by electrodeposition (Miyazaki et al. 2021). For example, metal sulfides have also been explored in developing sensors for ciprofloxacin detection, as demonstrated by Ali et al. (2023). They employed a Teflon-coated platinum electrode modified with polyaniline (PAni) and zinc sulfide (ZnS) nanocomposite (PAni-ZnS) (Fig. 3B). The detection limit of 0.50 µM was demonstrated. However, the sensor response validation using real samples was not carried out, which may limit its end use. Additionally, metal sulfides used as electrode material show poor interlayer spacing which results in restricted charge transfer, which may limit its commercial application (Barik and Ingole 2020).

## Biorecognition elements

In the context of CIP detection, biorecognition elements play a major role in identifying and interacting specifically with CIP. Most of the modifiers discussed above may be tuned to be specific to the class of quinolones but not CIP in particular. Recognition elements are biomolecules such as antibodies, aptamers, enzymes, and whole cells, which, upon interaction with the ciprofloxacin, generate a signal that is further used to quantify ciprofloxacin in the sample. To ensure efficient interaction between the bioreceptor and the analyte, managing the Debye length is crucial. Debye length can be optimized by controlling the ionic strength, maintaining optimal pH and temperature, or using spacers. Strategic positioning of the biorecognition elements using spacers such as mercapto hexanol and ethanolamine can improve the sensitivity and specificity of the sensor.

This section and SI-3 focus on some of the biorecognition elements used for developing electrochemical sensors for ciprofloxacin.

### Antibody

**Fig. 5:**
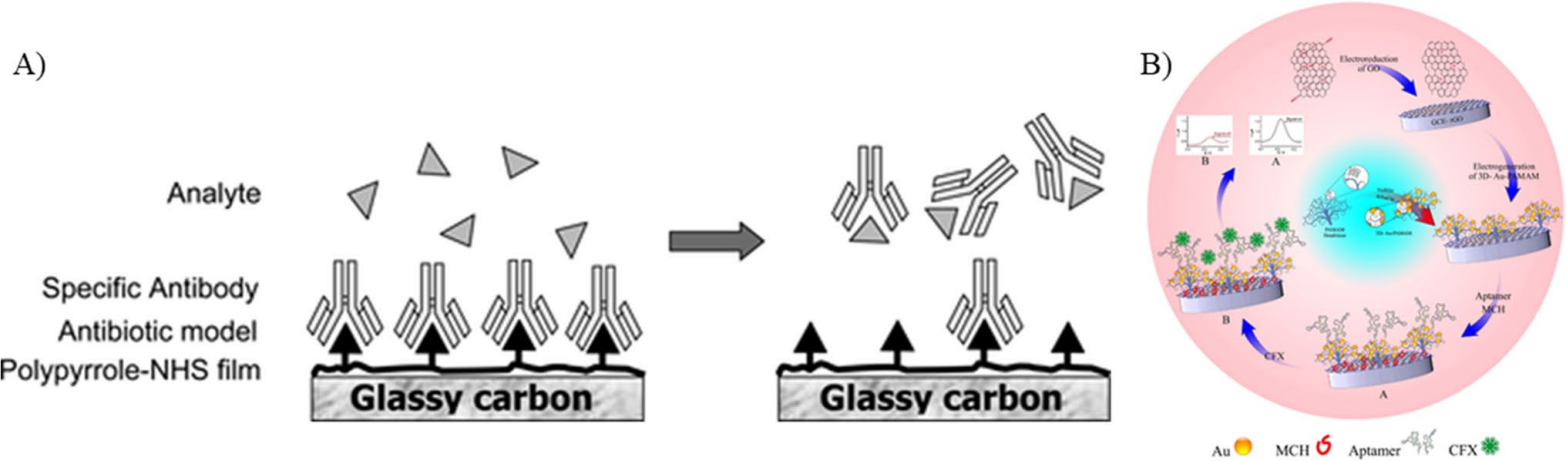
**A** Detection of ciprofloxacin based on the displacement of antibody in the presence of ciprofloxacin (reprinted with permission from Giroud et al. (2009), Copyright (2009), American Chemical Society). **B** Schematics of electrochemical detection of ciprofloxacin using aptamer as a biorecognition element (reprinted with permission from Mahmoudpour et al. (2021), Copyright (2021), Elsevier)

Antibodies, commonly used as biorecognition elements employed for their specificity and selectivity, are produced by injecting hapten-carrier protein into animals for stimulating an immune response. Monoclonal antibodies are more specific when compared to polyclonal antibodies (Majdinasab et al. 2020), but sometimes their in vitro production reduces the affinity (Lipman et al. 2005), which can be enhanced by screening strategies and targeted immunization (Nolli and Parenti 1991). Antibody-based biosensors have been extensively employed to detect ciprofloxacin. Ionescu et al. (2007) used impedance spectroscopy technique for detecting ciprofloxacin in serum and real blood samples. A modified gold-based substrate was employed, which was further modified with pyrrole-N hydroxy succinimide composite and incubated with polyclonal ciprofloxacin antibodies. To prevent non-specific binding of ciprofloxacin, 5% bovine serum albumin (BSA) was used. The interaction between the antibiotic and the antibody resulted in a reduction in the electrochemical signal, which was attributed to the hindered diffusion of redox molecules and the formation of layers on the electrode surface. Cyclic voltammetry studies revealed a limit of detection of 10.000 pg/mL. The biosensor shows high specificity to ciprofloxacin antibiotics. However, the study does not mention the operational stability of the biosensor, which is determined by the retention activity of the antibody used. This stability further determines the shelf life and reusability of the sensor. In a similar study conducted by Giroud et al. (2009), the working electrode of the immunosensor was modified with diamond paste before electro-polymerization with a polypyrrole-NHS composite. This deposited film was utilized for the covalent binding of the model antibiotic, ciprofloxacin, exploiting its amino group. Subsequently, the working electrode was incubated with anti-ciprofloxacin polyclonal antibodies. Detection of ciprofloxacin was correlated with the changes in impedance resulting from the displacement of antibody in the presence of CIP in the sample solution, as depicted in Fig. 5A. The sensor detected ciprofloxacin to extremely low concentrations of 1.00 pg/mL. Furthermore, the sensor showed potential for regeneration and reuse through a simple incubation process in an antibody solution. Even though this is a proof-of-concept study for ciprofloxacin detection, the sensitivity and specificity aspects of the biosensor were not studied. This can result in interferences by other antibiotics of the same class, which were also not studied. Though antibodies are specific to the analyte, their stability and shelf life are of great concern. They are susceptible to degradation under various environmental changes in pH and temperature. To address these issues, antibody can be encapsulated, chemically modified, or subjected to lyophilization. High cost of the antibodies can be resolved by using synthetic antibodies and optimizing the synthesis procedure.

### Aptamer

Aptasensors are the sensors developed by immobilizing aptamers on a substrate with suitable linker chemistry, altering the electron transport between the target analyte and the electrode. In a study conducted by Yang et al. (2021), a photoelectrochemical sensor was fabricated to detect ciprofloxacin in river water. Indium tin oxide (ITO) was used as the WE, which was further modified with Bi_24_O_31_Cl_10_/BiOCl heterojunction followed by immobilization of aptamer on it. The achieved detection limit was 1.67 ng/L (Yang et al. 2021). Although aptamers are highly specific and selective, they are easily prone to degradation due to factors such as pH change, high salt concentrations, and enzymatic cleavage. The degradation of aptamers might hinder their specificity and lead to false results. The presence of various interferences in the real samples might introduce non-specific binding, leading to inaccurate detection of the analyte. Mahmoudpour et al. (2021) detected ciprofloxacin in milk samples using a GCE modified with rGO, PAMAM, and gold nanoparticles (AuNPs). Further, NH_2_-aptamer was immobilized for specific analyte detection, as represented in Fig. 5B. The authors demonstrated a quantification limit of 1.00 nM. The sensitivity of the sensor can be further improved by reducing the Debye length. Despite the potential of aptamers in specific detection, they face challenges which impacts the effectiveness of the sensor. They are unstable under changes in pH and ionic strength, leading to conformational changes. This can be mitigated by chemically modifying the aptamer with phosphorothioate backbones. Additionally, phosphorothioate backbones act like nuclease-resistant modification. Synthesis of aptamers involves a process called Systematic Evolution of Ligands by Exponential Enrichment (SELEX), which is an expensive and labor-intensive process. However, the cost of this process can be reduced by using automated synthesis platform and inexpensive reagents.

### Molecularly imprinted polymers (MIP)

Molecularly imprinted polymers (MIPs) are artificial biorecognition elements where the monomers form noncovalent bonds with analyte in the presence of crosslinker and a solvent (Nag et al. 2024a). They are polymerized on the working electrode using simple techniques like cyclic voltammetry. The template (analyte) will be extracted after the polymerization, leaving behind the sites which are complementary to the target molecule. Despite their complementary structure, challenges such as non-specific binding and complete removal of the template exist. In a study conducted by Surya et al. (2020), a GCE modified with chitosan-gold nanoparticles (Ch-AuNP)-decorated MIP (Ch-AuMIP) was employed to detect ciprofloxacin. The amino groups and hydroxyl group chitosan may interact with CIP through electrostatic attraction and hydrogen bonding, helping in enhancing the binding efficiency of the MIP. Whereas, the AuNPs increase the conductivity, facilitating the transfer of electrons. Despite the advancements exhibited by Ch-AuMIP, as degradation or agglomeration of the nanoparticles can affect the sensor performance, stability of the nanoparticles over a long-term and differing environmental conditions can be a concern. Additionally, though the sensor offers good selectivity for CIP over other interferences, similar structured antibiotics present in a complex matrix may still interfere with the sensor performance. Though MIPs are promising recognition elements, they face several disadvantages that affect the sensor performance. One major drawback of the MIPs is their specificity and binding affinity. The imprinted sites may not completely accommodate all the variations in the structure of the analyte in different matrices. This issue can be addressed by optimizing the polymerization condition and considering the post-polymerization conditions to refine the imprinted sites. Another drawback is the stability and reproducibility of the MIP-based sensor. Inconsistencies in the distribution of the recognition sites can lead to variable sensor response through different devices. This can be mitigated by using highly pure reagents and standardizing the manufacturing procedure. Additionally, interference from real samples can affect the accuracy of the sensor.

## Remediation techniques

Developing a remediation technology for CIP is critical due to its persistence and bioaccumulation in the environment, leading to AMR and a harmful impact on flora and fauna. The combination of CIP detection and remediation signifies a potential approach in mitigating the impact of CIP on the environment. The removal of CIP is mainly by separation or degradation techniques. Separation is a remediation technique involving sorption (physical removal) of the antibiotic from the matrix. It is further divided into adsorption, membrane filtration, and ion exchange. Whereas degradation is a process that breaks down the target molecule into a less harmful compound. Degradation studies of CIP mainly focus on advanced oxidation processes such as photocatalysis, ozonation, and oxidation by Fenton’s reagent. This section summarizes various technologies reported for the remediation of CIP from the aqueous phase.

### Adsorption

**Fig. 6:**
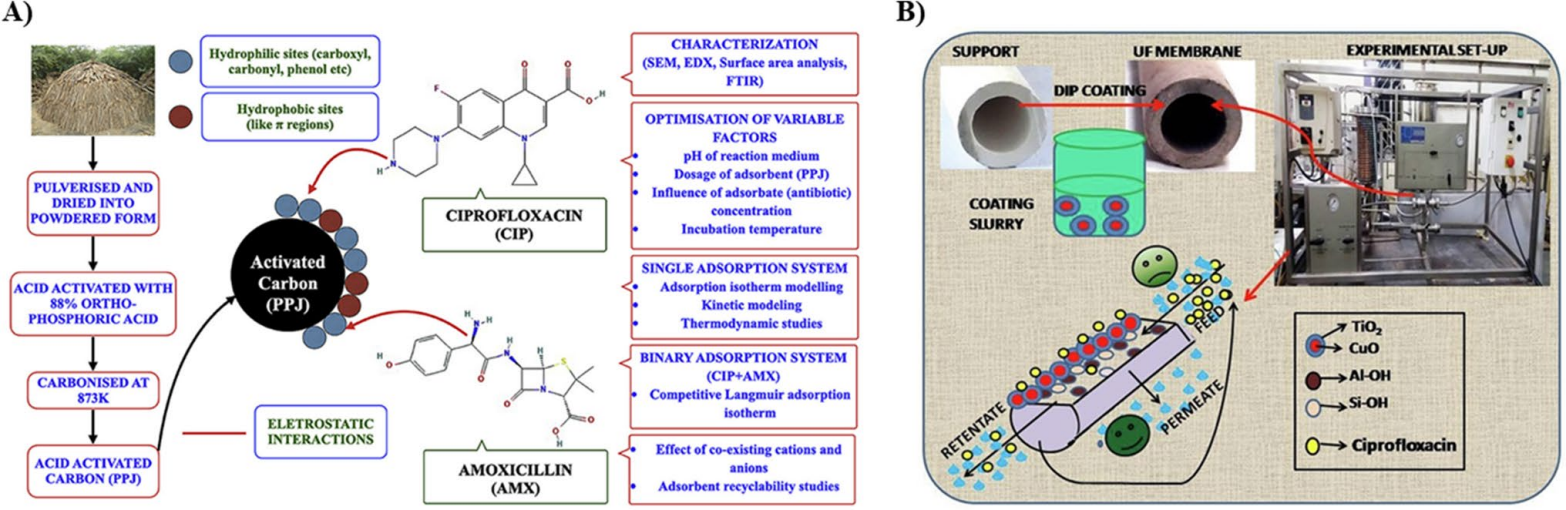
**A** Scheme representing synthesis of material and adsorption process (reprinted with permission from Chandrasekaran et al. (2020), Copyright (2020), Elsevier). **B** Representation of ciprofloxacin removal using membrane-based ultrafiltration (reprinted with permission from Bhattacharya et al. (2019), Copyright (2019), Elsevier)

Adsorption is a separation operation that is widely studied for antibiotic removal due to its efficiency and versatility. The material used for adsorption is called an adsorbent, and factors like pH, temperature, and antibiotic concentrations play a pivotal role in the efficacy of an adsorbent. Further improvement in efficiency and economy of the adsorption operation depends on the choice of the (Nayak et al. 2024). Hence, adsorbent material can be functionalized with ligands (Ali et al. 2019), biomolecules (Shao et al. 2019), or polymers (Patra and Narayanasamy 2022) to enhance the surface functionality and selectivity. Adsorption by activated carbon and biosorbents is widely employed for the CIP removal from the aqueous phase for their high surface area and tuneable pores which provide ample sites for antibiotic adsorption using activated carbon (AC). AC can be derived from various sources, such as coconut shells, plants, and other lignocellulosic materials which have shown higher adsorptive removal of antibiotics than most materials; however, challenges like saturation of the adsorption sites and competition with other ions and molecules exist. Similarly, biosorption is a process which utilizes biological materials like microorganisms or plant-based substances to remove antibiotics from the aqueous phase. This process involves the interaction between the functional groups present in the biological material and the antibiotic. Chandrasekaran et al. (2020) reported the synthesis of AC using *Prosopis juliflora* wood (PPJ) for adsorbing ciprofloxacin and amoxicillin from water. The physico-chemical alterations were studied, and the results revealed that the antibiotic formed a monolayer with PPJ through chemisorption. Enhanced adsorptive properties have been reported when using a combination of modified activated carbon with other adsorbents, such as MWCNTs (Fig. 6A). However, the practical implementation and scalability of the adsorbent for larger applications need to be thoroughly examined. In another study conducted by Fan et al. (2020), citric acid was used to modify banyan aerial roots and modified banyan roots were employed to remove ciprofloxacin. The modifications improved the removal capacity of the fiber; however, the experiments were conducted under specific conditions, possibly ignoring the impact of the different parameters in real-world scenarios. Further, the adsorption process was governed by pseudo-second-order adsorption kinetics and followed the Freundlich isotherm model. Moreover, to evaluate the sustainability and practical applicability of adsorption, the long-term stability and scalability of the biomaterial need to be assessed and quantified. Further description of adsorption is given in SI-4.

### Membrane filtration

Membrane filtration is one of the potential removal techniques studied for the removal of antibiotics. This pressure-driven technique employs semipermeable membranes with different pore sizes, such as ultrafiltration (Palacio et al. 2018), microfiltration (Yang et al. 2024), nanofiltration (Zaviska et al. 2013), and reverse osmosis (Alonso et al. 2018). The membrane filtration method involves selectively separating molecules based on their size, molecular weight, and charge by sieving, diffusion, and adsorption. In a study conducted by Bhattacharya et al. (2019), a novel CuO/TiO_2_ ceramic membrane was fabricated using copper oxide NP (CuO NP) in combination with TiO_2_ nanoparticles on a clay-alumina-based support. The rejection efficiency of the membrane was investigated, and 99.5% removal was observed in 60 min of operating time with a feed concentration of 500 µg/mL. Toxicity evaluation was also conducted on the treated solution by the membrane employing algae as an indicator. Further consideration of the selectivity of the membrane is important to avoid unintended removal of the beneficial components from the solution. The technique of single-step removal of CIP using nanocomposite-based ceramic ultrafiltration membrane was efficient, but long-term application would lead to membrane fouling. Moreover, the cost-effectiveness and scalability of the technique must be thoroughly investigated to enhance its applicability in the environment. A technique involving ultrafiltration membranes combined with water-soluble polymers has gained attention for its efficiency in removing small molecules from aqueous samples. Palacio et al. (2020) demonstrated the removal of amoxicillin, CIP, and tetracycline from an aqueous system using an ultrafiltration membrane conjugated with alkylated chitosan polyelectrolyte (ChA-PE). At pH 11, the retention percentage was 80% with respect to various molar ratios of antibiotics. The Cha-PE exhibited a retention capacity of 185.6 mg/g, 420.2 mg/g, and 632.8 mg/g for ciprofloxacin, amoxicillin, and tetracycline. Since the study focuses on a single pH, applying this technology to real-world problems is difficult since antibiotic-contaminated water sources will have varying pH.

### Ion exchange

Ion exchange is a technique which is used to separate the target molecules, where the ions in the solution are exchanged with the ions present in the solid phase (ion exchange resin). Cation exchange and anion exchange are the two commonly used methods, and parameters like pH, ion selectivity, and the need for periodic regeneration influence the efficiency of the process. Cation exchange is highly suitable for positively charged antibiotics, while anion exchange is suitable for negatively charged antibiotics. In research conducted by Li et al. (2024), the use of porous carbonized resins as precursors for synthesizing adsorbent material proved effective for ciprofloxacin removal. The precursor was derived from waste cationic exchange resin, and 93% antibiotic removal was reported with an adsorbent dosage of 300 mg/L. The adsorption kinetics followed a pseudo-second-order model, and adsorption thermodynamics revealed a spontaneous exothermic nature of the process. Langmuir and Freundlich isotherms were fitted to the experimental data, and the adsorption was better described by Langmuir isotherm, indicating monolayer adsorption. Even though the study shows efficient adsorption and reusability of the material, the process of regeneration of the material might increase the complexity and overall cost of the process. Additionally, scaling up the process to an industrial level from a laboratory level is crucial and may effect overall efficiency due to competitive adsorption from other pollutants. The study conducted by dos Santos Soldan et al. (2023) investigated the adsorption behavior of ciprofloxacin on the Amberlite IR120 (cationic resin). Kinetics and equilibrium studies were conducted to study the influence of agitation rate, pH, temperature, and resin mass on removal efficiency. Langmuir model was employed to determine the maximum adsorption capacity, which was 37 mg/g. The maximum removal was observed at a pH of 7, resin mass of 0.08 g, temperature of 15 °C, and an agitation rate of 300 rpm, and pseudo-first-order and pseudo-second-order kinetic models displayed the best fit for all the experimental results. Antibacterial effects of the treated sample were studied against *E. coli*, which proved the elimination of the antibiotic. However, it is necessary to analyze the performance of the material under diverse environmental factors and other pollutants to understand the potential interferences and effects of competitive adsorption caused by these interferences. Table 6 represents the list of different techniques used in the separation of ciprofloxacin, respectively.

**Table 6:** List of different techniques used in separation of ciprofloxacin

Method	Adsorbent	pH	Temperature (°C)	Adsorption capacity (mg/g)	Reference
Adsorption	Bentonite and chitosan composite	NA	30.0	39.06	Arya and Philip (2016)
	Fe_3_O_4_/carbon	7.0	30.0	90.10	Mao et al. (2016)
	Fe3O4/SiO2/Schiff base	5	NA	41.53 × 10^1^	Amirmahani et al. (2020)
Membrane filtration	Cellulose acetate nanofiber membrane impregnated with montmorillonite-	6	30	13.80	Das et al. (2020)
Ion exchange	Poly (acrylamide-co-itaconic acid)	6	28	17.85 × 10^1^	Bajpai and Bhowmik (2010)
	Montmorillonite-	-	NA	33.00 × 10^1^	Wang et al. (2010a)
	Polystyrene anion exchange resin + nanoconfined iron	5	25	96.20	Song et al. (2022)

### Photocatalysis

**Fig. 7:**
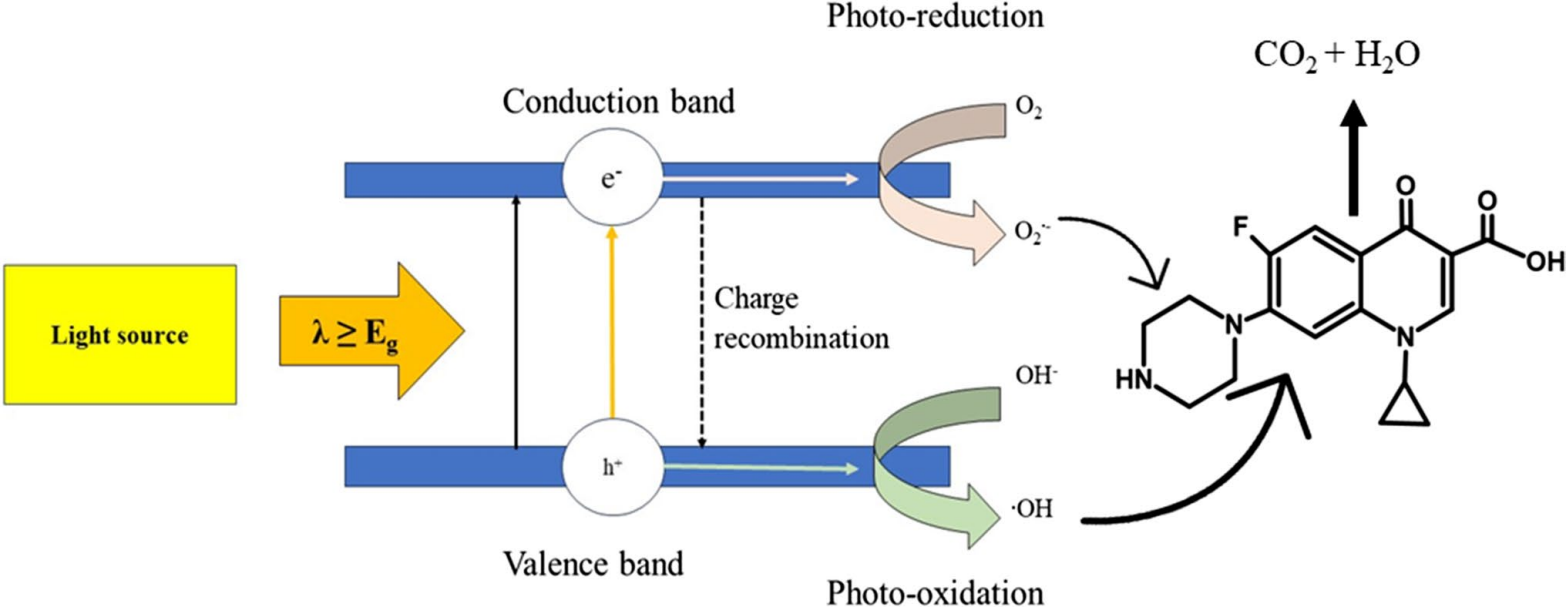
Scheme representing the basic principle of photodegradation (re-drawn using Microsoft PowerPoint)

Photocatalysts are specially designed compounds that absorb light with energy equal to or greater than their band gap and undergo electronic excitation, which results in electron–hole pair generation. At this stage, a photochemical reaction is initiated, in which the molecules combine with oxygen to generate reactive oxygen species (Fig. 7). The oxidative process also produces intermediate species, including free radicals, which are essential for starting a chain reaction that breaks or modifies the chemical structure of the target pollutant (Fig. SI-3). Semiconducting materials like titanium dioxide (Kutuzova et al. 2021), zinc oxide (Van Thuan et al. 2022), bismuth vanadate (Chen et al. 2018), graphitic carbon nitride (Chuaicham et al. 2021), and strontium titanate (Mohanty et al. 2023) are usually employed as photocatalysts in remediation. Gad-Allah et al. (2011) used titanium dioxide (TiO_2_) as a photocatalyst to degrade ciprofloxacin under simulated sunlight. It was observed that an increase in TiO_2_ concentration reduced the rate of reaction due to lower light transmittance. Degradation of the antibiotic by the photocatalyst was conducted at a pH of 5.8 and followed pseudo-first-order kinetics. While this is an efficient method for removing ciprofloxacin, the efficiency of the catalyst may decrease due to fouling and deactivation. Additionally, problems related to feasibility and cost exist, making the widespread adoption of this technique difficult. In a similar study involving semiconductor-assisted photocatalytic degradation, El-Kemary et al*.* used zinc oxide nanoparticles for the degradation of ciprofloxacin under the influence of irradiated UV light (El-Kemary et al. 2010). The photodegradation followed pseudo-first-order kinetics with efficient degradation observed at pH 7 and 10 which hindered its performance in real wastewater samples. Additionally, there is a requirement for a UV light source to activate the catalyst, raising concerns regarding the energy consumption and availability of UV light sources in real-world scenarios.

### Ozonation

**Fig. 8:**
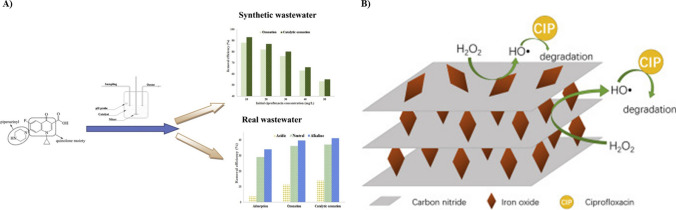
**A** Scheme representing ozonation in ciprofloxacin degradation (reprinted with permission from Nemati Sani et al. (2019), Copyright (2019), Elsevier). **B** Representation of ciprofloxacin degradation using dark Fenton’s process (reprinted with permission from Ding et al. (2019), Copyright (2019), Elsevier)

Ozonation is one of the most effective advanced oxidation processes (AOPs) in antibiotic remediation. Its capability lies in breaking down the antibiotic into a less harmful form. Ozone or hydroxyl radicals play a key role in this process. As a powerful oxidizing agent, ozone oxidizes the target antibiotic by attacking electron-rich moieties like double bonds or aromatic rings. This process is followed by the generation of hydroxyl ions, which further degrades the target antibiotic. The ozonation method shows high degradation rates, up to 90%. Aleksić et al. (2021) reported that ciprofloxacin and amoxicillin can be successfully removed using ozonation in an alkaline medium with degradation efficiency for ciprofloxacin of about 96%. Though the results demonstrated the decrease in toxicity of the wastewater after ozonation, the study employed model hospital wastewater, which might not completely represent the complex composition of real hospital wastewater. A wide range of interferences present in hospital wastewater might limit the use of the material in real-world scenarios. Nemati Sani et al. (2019) demonstrated catalytic ozonation using gama-Al_2_O_3_ nanoparticles to remove ciprofloxacin from synthetic wastewater and real wastewater (Fig. 8A). Rate of catalytic ozonation was highest at pH 9.5 and lowest at pH 5.25, and the degradation process was governed by pseudo-first-order kinetics. Even though this process degraded ciprofloxacin in both synthetic and real wastewater, the efficiency of CIP removal in the real wastewater was lower due to the scavenging effect of other pollutants, making the application of the material difficult in real-world scenarios.

### Oxidation by Fenton’s reagents

Fenton’s reaction is an oxidative process where hydroxyl radicals will be generated by the reaction between H_2_O_2_ and Fe^2+^ ions. The generated hydroxyl ions are highly reactive and oxidizing agents. It reacts with molecules by abstracting hydrogen atoms, leading to its degradation and making it less harmful. In a study by Wang et al. (2020), Fe^3+^ was inserted into the Nafion membrane to develop a Fenton catalyst, and the stability of the membrane was examined in the presence of UV. The process completely degraded ciprofloxacin within 4 h, and an efficiency of 97.7% was achieved within 3 h. A cyclic test was conducted, where the membrane showed an approximate degradation rate of 80%, and it decreased to about 20% after five cycles. The decrease in degradation rate can be due to the conversion of Fe^3+^ to Fe_3_O_4_. Ding et al. (2019) reported the synthesis of graphitic carbon nitride (g-C_3_N_4_)-iron oxide composite by in situ thermal condensation of dicyandiamide. The catalytic effect of the composite was studied in the dark Fenton system (Fig. 8B), and the system was studied with various parameters. From oxidation studies, mineralization of CIP was observed at pH 3, 0.0056 M H_2_O_2_, 1 g/L CN@IO-2, and 20 mg/L CIP. However, essential parameters which signify the practical application, such as stability and recyclability, were not studied, which can contribute to the possible application of the material in real-world conditions. In addition, the catalyst requires frequent replacement/reactivation upon inefficiency in recyclability and stability, leading to increased costs. Table 7 represents the list of various techniques used for the degradation of ciprofloxacin.

**Table 7:** List of different techniques used in separation of ciprofloxacin

Method	Adsorbent	Time	Efficiency (%)	Reference
Photocatalysis	ZnO + Ag_2_O	60 min	31.00	Zhao et al. (2017)
	Yttrium-doped bismuth oxy bromide	60 min	88.00	Imam et al. (2018)
	ZnO	140 min	10.00 × 10^1^	Eskandari et al. (2018)
Bioremediation	*Pleurotus ostreatus*	14 days	91.34 (methyl orange assay)	Singh et al. (2017)
	*Pycnoporus sanguineus*	2 days	98.50	Gao et al. (2018)
	Microalgae and bacteria consortium	NA	96.10 ± 0.07	Wang et al. (2023)
Ozonation	Gamma-Al_2_O_3_	60 min	93.00	Nemati Sani et al. (2019)
	Ozone	120 min	10.00 × 10^1^	Aleksić et al. (2021)
Fenton’s reaction	Graphitic carbon nitride	45 min	10.00 × 10^1^	Ding et al. (2019)
	OCNTs/FeOCl natural air cathode	90	10.00 × 10^1^	Liu et al. (2022)

### Bioremediation

**Fig. 9:**
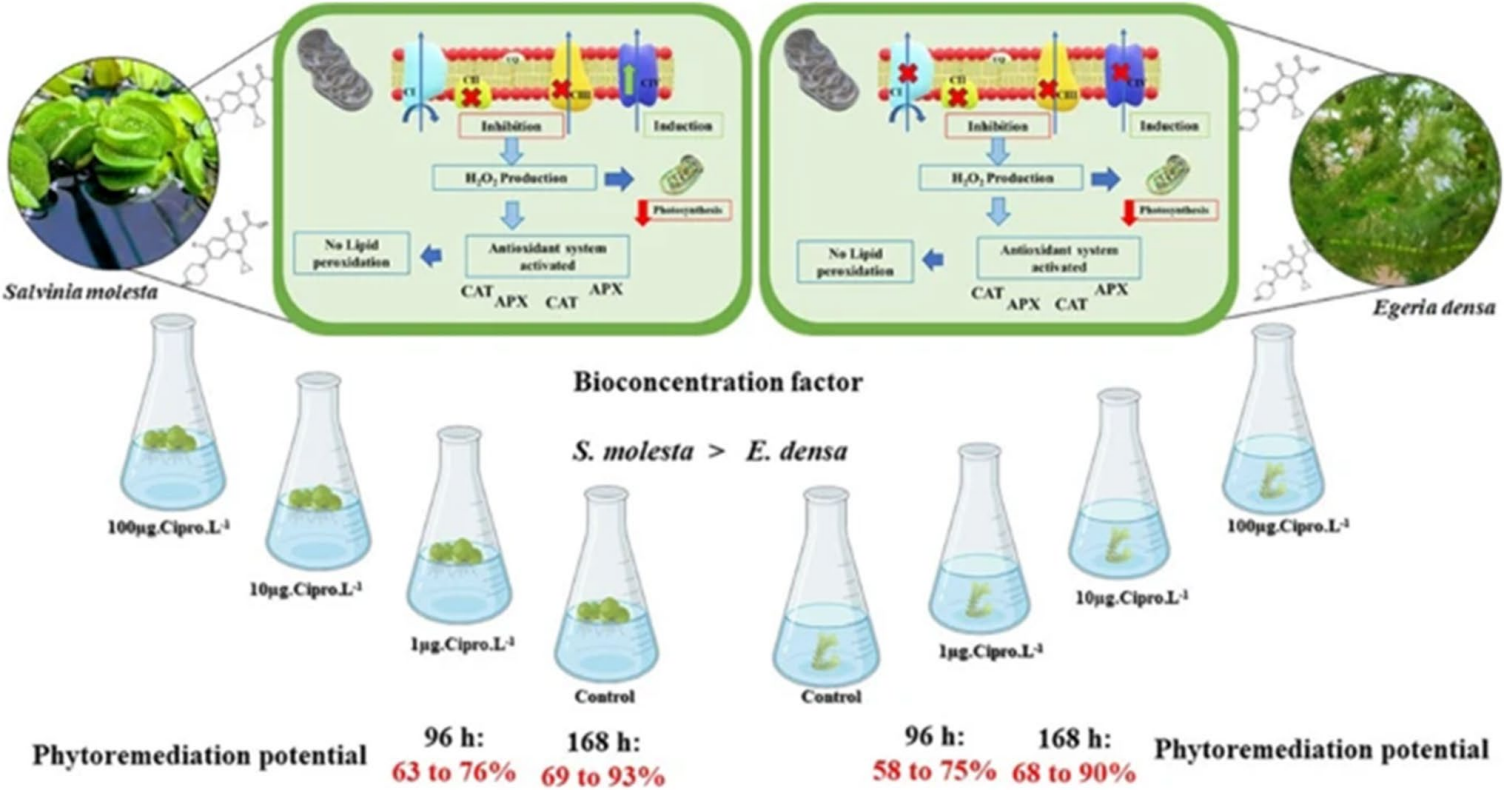
Scheme representing phytoremediation of ciprofloxacin using *S. molesta* and *E. densa* in artificially contaminated water (reprinted with permission from Kitamura et al. (2023), Copyright (2023), Springer Nature)

Bioremediation is an interplay of enzymatic reactions and microbial metabolic pathways aimed at the degradation or transformation of antibiotics present in the environment. Degradation of ciprofloxacin may be achieved using microbial degradation or by phytoremediation. Microbial degradation is a process in which microorganisms like bacteria and fungi play an important role in the degradation of antibiotics into less harmful forms. Similarly, enzymatic transformation involves the production of enzymes by microorganisms specifically tailored to degrade the antibiotic. The degraded compounds from bioremediation serve as a source of food for the microorganisms. Likewise, in phytoremediation, plants play a crucial role in absorbing, accumulating, and detoxifying antibiotics. Studies have demonstrated that plants and microorganisms absorb the antibiotics from soil and water and metabolize them within their tissues. Singh et al. (2017) used *Pleurotus ostreatus*, a basidiomycetous fungus, to study ciprofloxacin degradation, employing titrimetric analysis and spectrophotometric studies. The study was validated using HPLC, and the treated sample showed a lower antimicrobial activity. Though the method demonstrated considerable degradation potential, toxicity assays of the end products are important before the release into the environment and investigated the use of basidiomycetous fungi named *Pleurotus ostreatus* for ciprofloxacin degradation. Titrimetric analysis and spectrophotometric methods were used to evaluate the degradation of ciprofloxacin and were validated using HPLC and microbial inhibition studies. However, toxicity studies of the degraded products can be examined to mitigate the risks due to secondary environmental risks. A study conducted by Kitamura et al. (2023) employed *Salvinia molesta* D.S. Mitchell (floating plant) and *Egeria densa* Planch (submerged plant) for the remediation of ciprofloxacin in simulated polluted water. Both plants were exposed to an elevated concentration of ciprofloxacin for 96 h and 168 h, as depicted in Fig. 9. The plants exhibited the capability to absorb 58% of the antibiotic from the artificial media and showed greater accumulation of *S. molesta*; however, *E. densa* was observed to completely metabolized ciprofloxacin within its tissues. Moreover, the use of simulated contaminated water will lack complexity when compared to the environmental samples. Hence, the use of this technology is limited in effectively solving environmental problems. Additionally, the introduction of invasive species like *Salvinia molesta* might potentially affect the growth of native species and disturb aquatic life. Further description is given in the SI-5.

## Discussion and conclusion

Ciprofloxacin is one of the most extensively used fluoroquinolone antibiotics employed for treating intra-abdominal infections, skin infections, urinary tract infections, sexually transmitted diseases, and respiratory tract infections. However, administered CIP is partially metabolized in the body and is released into the environment through human excreta leading to the contamination of soil and water. These environments with sub-lethal concentrations of CIP act like a reservoir, contributing towards AMR. The proliferation and horizontal gene transfer in bacteria significantly increases antibiotic-resistant bacteria (ARBs), thereby reducing the effectiveness of standard therapies, increasing the cost of healthcare, complicating the treatment for bacterial infections, and contributing to high mortality rates.

The environmental risk caused by CIP can be evaluated by comparing its predicted environmental concentration (PEC) to predicted no-effect concentration (PNEC) value. If PEC exceeds PNEC, it suggests a potential risk to the environment and the proliferation of ARBs. A retrospective analysis in the year 2020 reported that over 34.9% of the global analysis for CIP exceeded the PNEC value (Booth et al. 2020). Another study reported that the concentration of CIP in Sirsa and Sutlej rivers exceeded the recommended levels by 1500 times (Gangar and Patra 2023). Also, resistance of *Escherichia coli* to fluoroquinolones increased from 78 to 85% between 2008 and 2013, and *Salmonella typhi* isolates also showed an increase in fluoroquinolone resistance from 8% in 2008 to 28% in 2014 (Laxminarayan and Chaudhury 2016). Similarly, 36% of multidrug-resistant *Mycobacterium tuberculosis* complex strains showed fluoroquinolone resistance in 2022, contributing to high pre-extensive drug resistance in Mumbai, India (Dreyer et al. 2022).

The ability of ciprofloxacin to resist the degradation process in municipal effluent treatment plants and hospital effluent treatment plants poses a challenge in complete removal. Additionally, the lack of point-of-use technologies, preloaded remediation, and lack of advanced automation technology of regeneration have further affected the removal of CIP from the environment. To overcome the problems caused by CIP, simulation study can help in the precise modelling of the detection and removal of the antibiotic. It helps by optimizing the treatment strategy, assessing the byproducts, and designing an inexpensive scalable solution for the detection and mitigation of CIP from the environment.

In the development of a sensor, the first step of choosing an electrochemical substrate is based on factors such as sensitivity, portability, and cost, with SPE being preferred for ease of manufacturing, mass fabrication, portability, and robustness. To enhance the electrocatalytic activity and sensitivity of the sensor, the chosen substrate is further modified with nanomaterials such as graphene, CNTs, and metal oxides, using suitable linker chemistries. Recent advancements in MOFs, COFs, and quantum dots have displayed promising results in CIP detection. Additionally, to increase the specificity of the sensor, biorecognition elements like aptamer or antibody are immobilized on the modified substrate using a suitable bioconjugation technique. The last step in designing a sensor is the conversion of the electrochemical signals to readable outputs (Fig. SI-4).

Remediation of antibiotics in effluent treatment plants plays a critical role in addressing fluoroquinolone resistance by limiting the co-existence of the drug and bacteria in aquatic pools. Despite the advantages offered by the conventional adsorption-based remediation technique, there exist challenges such as regeneration issues and loss of efficiency with time. Other techniques are being explored in research, but they face challenges for real-world application. For example, membrane filtration faces fouling due to other contaminants in the matrix, photocatalysis encounters limited effectiveness under low light, and ozonation may produce harmful byproducts such as bromates and perchlorates. Advanced processes incorporating Fenton’s reactions may further increase the toxicity levels in sludge due to the formation of peroxides and chlorinated compounds. Also, bioremediation is often restrained by the efficiency of plants and microorganisms.

Combining ciprofloxacin detection and remediation signifies a potential approach to mitigating the impact of ciprofloxacin on the environment. The widespread adoption of biosensors and remediation technologies holds a robust and ecologically balanced future. By prioritizing and practically applying these technologies, there can be a significant reduction of the contamination caused by ciprofloxacin, thus fostering sustainable co-existence with the antibiotics in the environment. Advanced technologies like point-of-care tools are promising strategies for obtaining more accurate and timely detection of infections caused by pathogens, enabling healthcare professionals to prescribe specific antibiotics. Moreover, the development of effective remediation strategies provides potential solutions for combating antimicrobial resistance. Integration of these technologies in healthcare promotes better results with minimum risk of developing resistance towards the antibiotic.

## Supplementary Information

Below is the link to the electronic supplementary material.

Supplementary file1 (DOCX 601 KB)

## Data Availability

All the required data are provided in the manuscript and supplementary information.
