# Supplementary material for: Recent advances in electrochemical sensing and remediation technologies for ciprofloxacin
Source: Environ Sci Pollut Res Int. 2025 Jan 14;32(5):2210–37. doi: 10.1007/s11356-024-35852-9 (PMC11802654; doi:10.1007/s11356-024-35852-9)
Supplement: Supplementary file 1 — Supplementary file1 (DOCX 601 KB) [file 11356_2024_35852_MOESM1_ESM.docx]

**Supporting Information (SI)**

**SI-1 Structure and action of ciprofloxacin**

Ciprofloxacin (CIP) has a quinoline-4(1H)-one backbone, constituting a bicyclic aromatic structure. Specifically, it includes a cyclopropyl group at the first position, a carboxylic acid attached to the third position, a fluorine substituent at the sixth position and a piperazine-1-yl substituent at the seventh position (Vardanyan and Hruby, 2006). It is bactericidal in action and inhibits DNA replication by inhibiting enzymes like topoisomerase II and IV. Low concentrations of CIP initially results in bacteriostasis and finally leads to cell death.

**SI-2 Modifiers**

**SI-2.1 Graphene**

Graphene is made up of a monolayer of sp^2^ hybridized carbon atoms arranged in a hexagonal lattice. As a 2D material, it effectively has all its delocalized π-conjugated electrons on its surface. The electronic properties, high surface area, and electrical conductivity make graphene an ideal modifier in sensing applications.

**SI-2.2 Carbon nanotubes**

Another popular nanomaterial which is extensively used in sensing applications is carbon nanotubes (CNTs). CNTs appear as seamless hollow tubes made of rolling graphite sheet and can be separated into single-walled carbon nanotubes (SWCNTs) and multi-walled carbon nanotubes (MWCNTs) based on the number of layers in the graphite sheet (Yang et al., 2015).

**SI-2.3 Covalent Organic Framework**

Covalent organic frameworks (COFs) are crystalline polymeric materials defined by excellent stability and long-range order. COFs are formed through the direct linkage of organic units via covalent bonding, eliminating the need for a metallic ion. Solvothermal (Feng et al., 2022) and ionothermal (Guan et al., 2018) methods are the most used techniques in COF synthesis. Based on the dimensions of the building blocks employed in constructing the COF, it can be classified into two-dimensional (2D) and three-dimensional (3D) COFs. 2D COFs have a layered structure resembling graphene. They consist of a π-column structure, which promotes carrier transport in the direction of stacking, making them eligible materials in photovoltaics and optoelectronics (Yang et al., 2020). Whereas 3D COFs consist of sp_3_ hybridized carbon/silicon atom, which connects the monomers to form a network-like structure (Wang and Zhuang, 2019). High surface area, low density, and many open channels are some advantages of 3D COFs in sensing applications (Xie et al., 2020).

**SI-3 Biorecognition Elements**

**SI-3.1 Antibody**

Antibodies are commonly utilized as biorecognition elements in antibiotic screening due to their high specificity and selectivity. Antibiotics, being small molecules, lack inherent antigenicity, necessitating the production of antibodies against them. This is achieved by injecting hapten-carrier protein complexes into animals, thereby stimulating an immune response to produce specific antibodies.

**SI-3.2 Aptamer**

The functional biomolecules known as aptamers are chosen by an in vitro procedure known as SELEX. Aptamers are an excellent choice for bioreceptors because of their high affinity for their target. An aptasensor is a sensor that has been developed by immobilising aptamers on a substrate utilising suitable linker chemistry. When an aptamer binds to a particular analyte, it folds its single-stranded flexible chain into a defined 3D form. The difference in electron transport between the electrode and the analyte can be used to identify the aptamer-target complex that has formed.

### **SI-4 Adsorption**

Adsorption is a type of separation operation that is widely used in antibiotic removal due to its efficiency and versatility. Factors like pH, temperature and antibiotic concentrations play a pivotal role in the process of adsorption. The material used for adsorption is called an adsorbent. The efficiency and economy of the adsorption operation depend on the choice of the adsorbent. A wide range of materials, including raw lignocellulosic biomass, activated carbon, zeolites, clays, dead algal and fungal biomass and hybrid materials like polymeric nanocomposites and metal-organic frameworks, are explored as adsorbents. Adsorbent material can also be functionalized with ligands (Ali et al., 2019), biomolecules (Shao et al., 2019) or polymers (Patra and Narayanasamy, 2022) to enhance the surface functionality. Adsorption by activated carbon and biosorbents is most employed for the CIP removal from the aqueous phase.

#### **SI-4.1 Adsorption by activated carbon**

High surface area and tuneable pores provide ample sites for antibiotic adsorption using activated carbon (AC). Adsorption on activated carbon occurs due to forces like Van der Waals interaction, pi-pi interactions and hydrogen bonding. Activated carbon can be derived from various sources, such as coconut shells, plants and other lignocellulosic materials. It can be regenerated using desorption of the adsorbed antibiotics using thermal treatment or chemical regeneration techniques. Though it shows higher adsorption efficacy, challenges like saturation of the adsorption sites and competition with other ions and molecules exist. In another study, Arunkumar *et al.* (Chandrasekaran et al., 2020) reported the synthesis of AC using *Prosopis juliflora* wood for adsorbing ciprofloxacin and amoxicillin from water. *P. juliflora* was treated with acid, followed by pyrolysis to develop acid-activated carbon (PPJ). The methodology involved optimization of pH, dosage of PPJ, concentration of antibiotic and incubation temperature. Physico-chemical alterations occurring due to acid activation and adsorption were studied. The results revealed that the antibiotic formed a monolayer with PPJ through chemisorption, which was confirmed by Langmuir isotherm. The adsorbent material was regenerated and reused for 4 cycles without a considerable decrease in its adsorption efficiency. The assessment of surface modifications resulting from acid activation and subsequent adsorption on the PPJ surface involved evaluating past physico-chemical alterations. Enhanced adsorptive properties have been reported when using a combination of modified activated carbon with other adsorbents, such as MWCNTs. This combination has been found to be more effective in the removal of ciprofloxacin from the environment (Fig. 6 A). However, the practical implementation and scalability of the adsorbent for larger applications need to be thoroughly examined. Narges *et al.* (Sharifpour et al., 2020) developed an adsorbent composed of multi-walled nanotubes/activated carbon for ciprofloxacin removal. The study focused on optimizing factors such as time, temperature and adsorbent dosage to increase the efficiency of ciprofloxacin removal. The material could remove up to 73% of the drug from the water sample. The adsorption data favoured Freundlich isotherm with a good fit. Kinetic studies confirmed that the pseudo-second-ordered kinetics represented the adsorption process. Though the adsorbent could remove the antibiotic up to 73%, further use of sewage samples might help to model the real-world scenario. The complexity of the composition of the sewage might impact the efficiency of the adsorbent material. To evaluate the practicality of the developed material, further discussion regarding the scalability, long-term stability, toxicity of the developed material and cost-effectiveness is crucial.

#### **SI-4.2 Biosorption**

Biosorption is a process which utilizes biological materials like microorganisms or plant-based substances to remove antibiotics from the aqueous phase. This process involves the interaction between the functional groups present in the biological material and the antibiotic. The efficiency of this process is significantly influenced by two crucial factors: pH and temperature. Though this process is eco-friendly and cost-effective, drawbacks include the non-availability of abundant biosorbents and lesser efficiency compared to conventional adsorbents. Kristina *et al.* (Tolić et al., 2021) used maple leaves, tangerine peel, and tomato waste as adsorbents for the removal of ciprofloxacin. Maple leaves, tangerine peel and tomato waste samples were washed, air-dried, and subsequently oven-dried at 50°C before being hand-milled. The experimental results were described using Freundlich and Dubinin-Radushkevich modelling. Maple leaves showed the highest sorption, while tangerine peel showed weak sorption affinity. Though the synthesized adsorbent material showed potential in ciprofloxacin removal, the effectiveness of the technique is based on pH, ionic strength, temperature and the dosage of the synthesized material, making it crucial to achieve predictable and consistent results across varied conditions. Furthermore, biosorption is a cost-effective technique compared to conventional removal. However, the scalability and practicality of using the adsorbent synthesized from maple leaves, tangerine peel and tomato waste for large-scale application in water treatment plants are challenging. In a recent study conducted by Huimin et al. (Fan et al., 2020), banyan aerial roots were modified to remove ciprofloxacin. Banyan roots were modified using the thermochemical method. Citric acid was used to modify the roots, enhancing the fibre's capacity. The pseudo-second-order kinetic equation and Freundlich model exhibited a better fit with the experimental results. However, the study investigates the adsorption of ciprofloxacin in-depth; the experiments were conducted under specific conditions, possibly ignoring the impact of the different parameters in real-world scenarios. To evaluate sustainability and practical applicability, the long-term stability of the developed biomaterial needs to be assessed. Moreover, the feasibility of large-scale production in relation to real-world problems needs to be further investigated.

### **SI-5 Bioremediation**

Bioremediation is an interplay of enzymatic reactions and microbial metabolic pathways aimed at the degradation or transformation of antibiotics present in the environment. Microorganisms capable of degrading antibiotics deploy enzymes to break down the antibiotic. The pathways employed by the microorganisms result in disintegrating the antibiotic into simpler and non-harmful forms. Removal of ciprofloxacin may be achieved using microbial degradation or by phytoremediation.

#### **SI-5.1 Microbial degradation**

Microbial degradation is a process in which microorganisms like bacteria and fungi play an important role in the degradation of antibiotics into less harmful forms. The enzymatic transformation involves the production of enzymes by the microorganisms that are specifically tailored to degrade the antibiotic. Depending on the chemical structure of the antibiotic, microbial degradation includes diverse mechanisms like hydrolysis, oxidation, reduction and conjugation. The degraded compounds serve as a source of food for the microorganisms. Sushil et al. (Singh et al., 2017) in the year 2017, investigated the use of basidiomycetous fungi named *Pleurotus ostreatus* for ciprofloxacin degradation. Titrimetric analysis and spectrophotometric methods were used to determine the degradation of ciprofloxacin. In the spectrophotometric method, the Indigo carmine and methyl orange method were employed. Remediation of CIP was validated using HPLC and by studying the antimicrobial activity of the degraded products. The fungi showed a considerable degradation potential towards ciprofloxacin. The degraded products showed lower antimicrobial activity, which confirmed the degradation of ciprofloxacin. However, toxicity studies of the degraded products should have been conducted to mitigate the risks due to secondary environmental risks.

#### **SI-5.2 Phytoremediation**

Phytoremediation is an eco-friendly method employed in the degradation of antibiotics using plants. In this method, plants play a crucial role in absorbing, accumulating and detoxifying the antibiotics. Studies have demonstrated that plants absorb the antibiotics from soil and water through their roots, and they subsequently metabolize them within their tissues. These processes involve enzymatic degradation, microbial interactions in the rhizosphere and translocation in the plant species. In 2021, Theresa et.al (Adesanya et al., 2021), reported the use of *Typha latifolia* and *Panicum virgatum L* for the remediation of ciprofloxacin and sulfamethoxazole. At environmentally relevant concentrations, the uptake of the antibiotics by the plants was studied. Every 3-4 days, plants were destructively sampled during the 21-day growth period. It was observed that the accumulation of antibiotics was greater in the root than in other parts. Percentage uptake of the antibiotic was higher in *Typha latifolia* when compared to *Panica virgatum*. However, the use of a hydroponic growth system rather than the soil system might not completely provide insights into the complexity of interactions between the plant and the contaminant in the environment. In a similar study, Rafael *et.al* (Kitamura et al., 2023) employed *Salvinia molesta* D.S Mitchell (floating plant) and *Egeria densa* Planch (submerged plant) for the remediation of ciprofloxacin in artificially contaminated water. Both plants were exposed to an elevated concentration of ciprofloxacin for 96 hours and 168 hours, as depicted in Fig. 9. After 96 hours, the plants exhibited the capability to absorb 58% of the antibiotic from the artificial media. Though the phytoremediation efficiency remained consistent among the plants, *S. molesta* showed greater accumulation, while *E.densa* metabolized ciprofloxacin within its tissues. Also, the usse of simulated contaminated water will lack complexity when compared to the environmental samples. Hence, limiting the use of this technology in effectively solving environmental problems. Additionally, the introduction of invasive species like *Salvinia molest*a might potentially affect the growth of native species and disturb aquatic life.

**SI-6 Common methods used in the estimation of detection limit**

Limit of detection (LoD) is the lowest concentration of an analyte that can be detected. It is also determined as the signal that is three times the noise (S/N=3). LoD can be estimated using following formulae:

1. The formula 3 σ/M is a common method used in the estimation of the detection limit.

Where,

1. σ represents the standard deviation of the blank. Blank is a solution which contains no analyte. Measurements are taken with the blank solution to calculate the standard deviation (σ), which helps in quantifying the noise. The lower values of standard deviation of the blank (σ) represents that the sensor can detect lower analyte concentrations.
2. M represents the slope of the calibration chart. Calibration chart can be obtained by measuring the signals at various analyte concentrations. Slope can be calculated using the following formula:

$$M=\frac{\Delta signal}{Concentration of the analyte}$$

It is used to understand the change in the signal for each unit change in the concentration of the analyte. the higher slope values represents the higher sensitivity of the sensor, resulting in lower detection limit.

1. Any signal greater than X+3 σ can be considered as limit of detection.

Where,

1. X represents the average value of the blank signal. Measurements will be taken multiple times and the mean will be calculated.
2. σ represents the standard deviation of the blank measurements. It defines the variability in the background signal. Higher standard deviation represents more variability in the baseline measurements.
3. Any signal greater than 3σ can be considered as the limit of detection.

Where,

1. σ is the standard deviation, calculated by taking multiple measurements of blank.

**Figures**


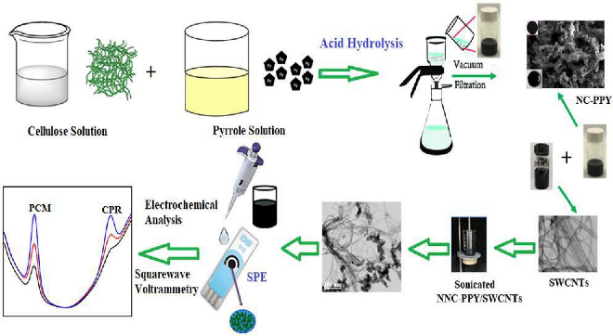


Fig. SI-1 Schematic representation of ciprofloxacin detection using a screen-printed electrode modified with nanocellulose-polypyrrole matrix and single walled carbon nanotube (Reprinted with permission from (Shalauddin et al., 2022), Copyright (2022), Elsevier).


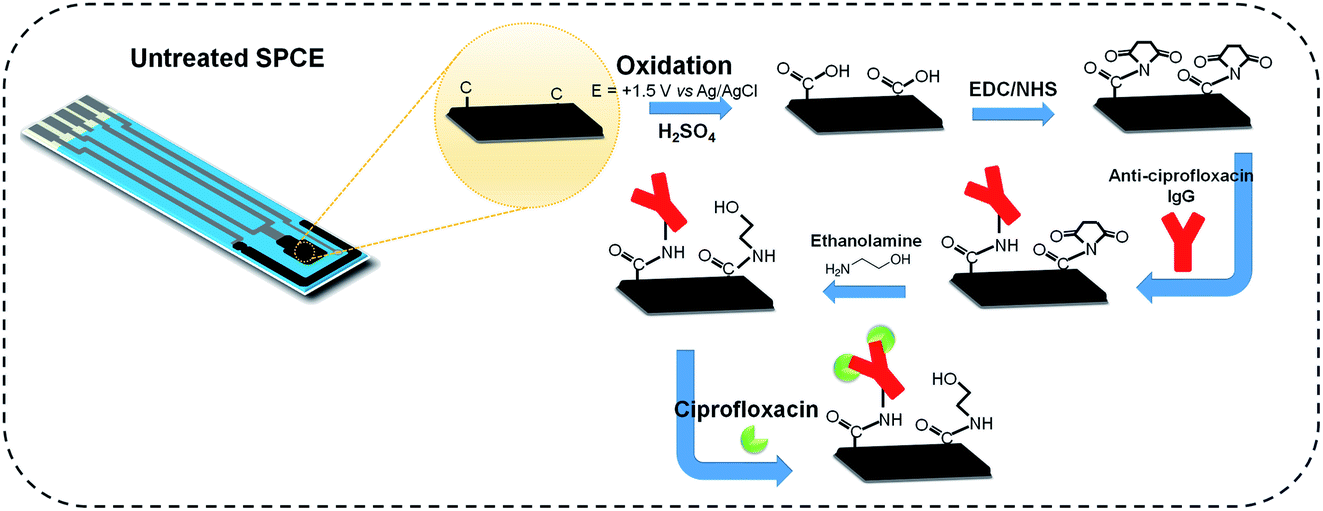


Fig. SI-2 Stepwise modification of the screen-printed electrode for detection of ciprofloxacin (Reprinted with permission from (Lamarca et al., 2020), Copyright (2020), RSC Advances).


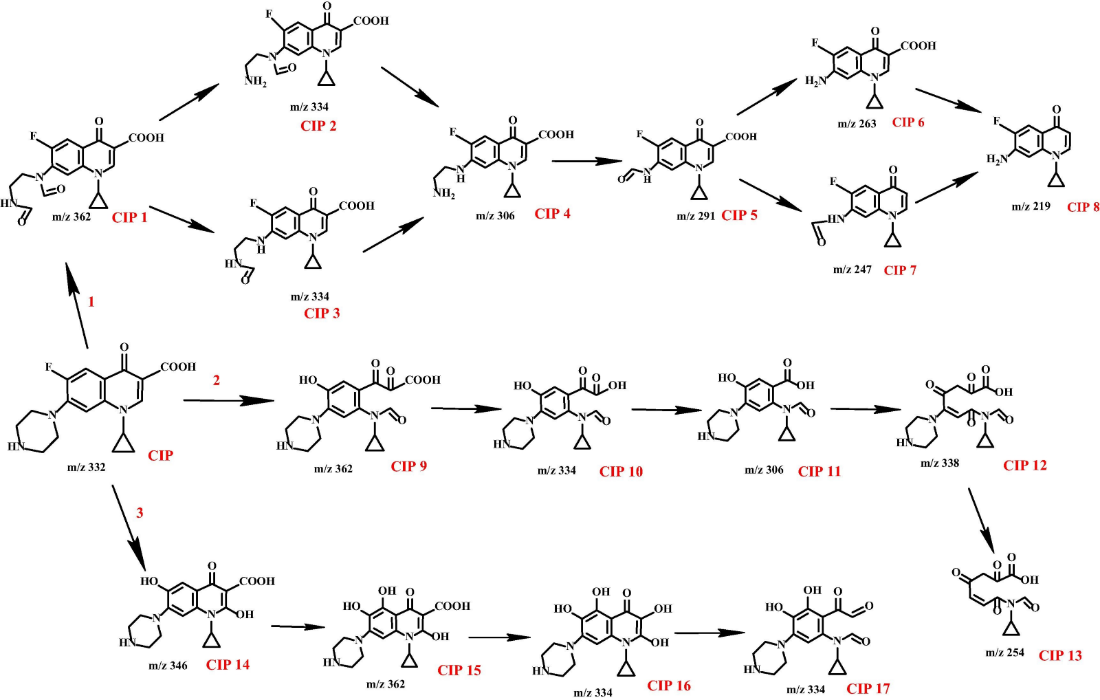


Fig. SI-3 Scheme representing photocatalytic degradation of ciprofloxacin (Reprinted with permission from (Wen et al., 2018), Copyright (2018), Elsevier)

**
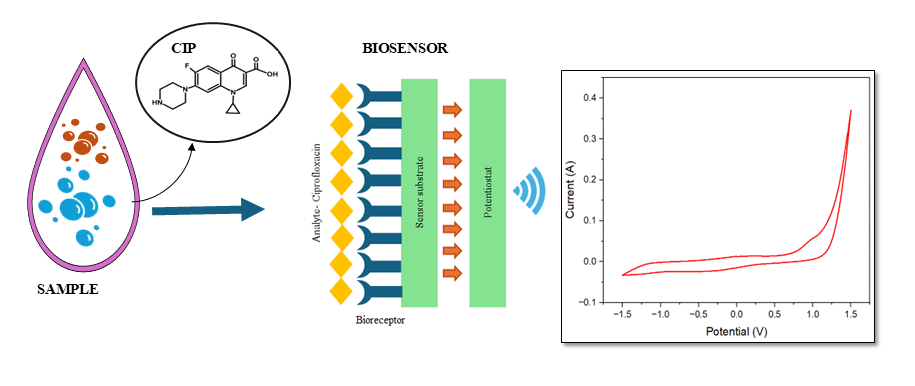
**

Fig. SI-4 Schematics of design of electrochemical biosensor.

**References**

Adesanya, T., Zvomuya, F., Farenhorst, A., 2021. Phytoextraction of ciprofloxacin and sulfamethoxaxole by cattail and switchgrass. Chemosphere 279, 130534. https://doi.org/10.1016/j.chemosphere.2021.130534

Ali, S.N.F., El-Shafey, E.I., Al-Busafi, S., Al-Lawati, H.A.J., 2019. Adsorption of chlorpheniramine and ibuprofen on surface functionalized activated carbons from deionized water and spiked hospital wastewater. J Environ Chem Eng 7, 102860. https://doi.org/10.1016/j.jece.2018.102860

Chandrasekaran, A., Patra, C., Narayanasamy, S., Subbiah, S., 2020. Adsorptive removal of Ciprofloxacin and Amoxicillin from single and binary aqueous systems using acid-activated carbon from Prosopis juliflora. Environ Res 188, 109825. https://doi.org/10.1016/j.envres.2020.109825

Feng, J., Zhang, Y.-J., Ma, S.-H., Yang, C., Wang, Z.-P., Ding, S.-Y., Li, Y., Wang, W., 2022. Fused-Ring-Linked Covalent Organic Frameworks. J Am Chem Soc 144, 6594–6603. https://doi.org/10.1021/jacs.2c02173

Guan, X., Ma, Y., Li, H., Yusran, Y., Xue, M., Fang, Q., Yan, Y., Valtchev, V., Qiu, S., 2018. Fast, Ambient Temperature and Pressure Ionothermal Synthesis of Three-Dimensional Covalent Organic Frameworks. J Am Chem Soc 140, 4494–4498. https://doi.org/10.1021/jacs.8b01320

Kitamura, R.S.A., Brito, J.C.M., Silva de Assis, H.C., Gomes, M.P., 2023. Physiological responses and phytoremediation capacity of floating and submerged aquatic macrophytes exposed to ciprofloxacin. Environmental Science and Pollution Research 30, 622–639. https://doi.org/10.1007/s11356-022-22253-z

Lamarca, R.S., Faria, R.A.D. de, Zanoni, M.V.B., Nalin, M., Lima Gomes, P.C.F. de, Messaddeq, Y., 2020. Simple, fast and environmentally friendly method to determine ciprofloxacin in wastewater samples based on an impedimetric immunosensor. RSC Adv 10, 1838–1847. https://doi.org/10.1039/C9RA09083E

Patra, C., Narayanasamy, S., 2022. Polypyrrole complexation on biomass-derived powdered carbon for adsorptive elimination of emerging pharmaceutical contaminant Sulfamethoxazole: A comprehensive insight. J Clean Prod 370, 133565. https://doi.org/10.1016/j.jclepro.2022.133565

Shalauddin, M., Akhter, S., Jeffrey Basirun, W., Sanghiran Lee, V., Rafie Johan, M., 2022. A metal free nanosensor based on nanocellulose-polypyrrole matrix and single-walled carbon nanotube: Experimental study and electroanalytical application for determination of paracetamol and ciprofloxacin. Environ Nanotechnol Monit Manag 18, 100691. https://doi.org/10.1016/j.enmm.2022.100691

Shao, B., Liu, Z., Zeng, G., Liu, Yang, Yang, X., Zhou, C., Chen, M., Liu, Yujie, Jiang, Y., Yan, M., 2019. Immobilization of laccase on hollow mesoporous carbon nanospheres: Noteworthy immobilization, excellent stability and efficacious for antibiotic contaminants removal. J Hazard Mater 362, 318–326. https://doi.org/10.1016/j.jhazmat.2018.08.069

Sharifpour, N., Moghaddam, F.M., Mardani, G., Malakootian, M., 2020. Evaluation of the activated carbon coated with multiwalled carbon nanotubes in removal of ciprofloxacin from aqueous solutions. Appl Water Sci 10, 140. https://doi.org/10.1007/s13201-020-01229-9

Tolić, K., Mutavdžić Pavlović, D., Stankir, N., Runje, M., 2021. Biosorbents from Tomato, Tangerine, and Maple Leaves for the Removal of Ciprofloxacin from Aqueous Media. Water Air Soil Pollut 232, 218. https://doi.org/10.1007/s11270-021-05153-9

Vardanyan, R.S., Hruby, V.J., 2006. Antimicrobial Drugs, in: Synthesis of Essential Drugs. Elsevier, pp. 499–523. https://doi.org/10.1016/B978-044452166-8/50033-9

Wang, J., Zhuang, S., 2019. Covalent organic frameworks (COFs) for environmental applications. Coord Chem Rev 400, 213046. https://doi.org/10.1016/j.ccr.2019.213046

Wen, X.-J., Niu, C.-G., Zhang, L., Liang, C., Guo, H., Zeng, G.-M., 2018. Photocatalytic degradation of ciprofloxacin by a novel Z-scheme CeO2–Ag/AgBr photocatalyst: Influencing factors, possible degradation pathways, and mechanism insight. J Catal 358, 141–154. https://doi.org/10.1016/j.jcat.2017.11.029

Xie, Y., Zhang, T., Chen, Y., Wang, Y., Wang, L., 2020. Fabrication of core-shell magnetic covalent organic frameworks composites and their application for highly sensitive detection of luteolin. Talanta 213, 120843. https://doi.org/10.1016/j.talanta.2020.120843

Yang, N., Chen, X., Ren, T., Zhang, P., Yang, D., 2015. Carbon nanotube based biosensors. Sens Actuators B Chem 207, 690–715. https://doi.org/10.1016/j.snb.2014.10.040

Yang, Q., Luo, M., Liu, K., Cao, H., Yan, H., 2020. Covalent organic frameworks for photocatalytic applications. Appl Catal B 276, 119174. https://doi.org/10.1016/j.apcatb.2020.119174
